# Structural and kinetic considerations on the catalysis of deoxyarbutin by tyrosinase

**DOI:** 10.1371/journal.pone.0187845

**Published:** 2017-11-14

**Authors:** Antonio Garcia-Jimenez, Jose Antonio Teruel-Puche, Pedro Antonio Garcia-Ruiz, Adrian Saura-Sanmartin, Jose Berna, Francisco Garcia-Canovas, José Neptuno Rodriguez-Lopez

**Affiliations:** 1 GENZ-Group of Research on Enzymology, Department of Biochemistry and Molecular Biology-A, Regional Campus of International Excellence "Campus Mare Nostrum", University of Murcia, Espinardo, Murcia, Spain; 2 Group of Molecular Interactions in Membranes, Department of Biochemistry and Molecular Biology-A, University of Murcia, Espinardo, Murcia, Spain; 3 Group of Chemistry of Carbohydrates, Industrial Polymers and Additives, Department of Organic Chemistry, Faculty of Veterinary, University of Murcia, Espinardo, Murcia, Spain; 4 Group of Synthetic Organic Chemistry, Department of Organic Chemistry, Faculty of Chemistry, University of Murcia, Espinardo, Murcia, Spain; Universidade Nova de Lisboa Instituto de Tecnologia Quimica e Biologica, PORTUGAL

## Abstract

Deoxyarbutin, a potent inhibitor of tyrosinase, could act as substrate of the enzyme. Oxytyrosinase is able to hydroxylate deoxyarbutin and finishes the catalytic cycle by oxidizing the formed *o*-diphenol to quinone, while the enzyme becomes deoxytyrosinase, which evolves to oxytyrosinase in the presence of oxygen. This compound is the only one described that does not release *o*-diphenol after the hydroxylation step. Oxytyrosinase hydroxylates the deoxyarbutin in *ortho* position of the phenolic hydroxyl group by means of an aromatic electrophilic substitution. As the oxygen orbitals and the copper atoms are not coplanar, but in axial/equatorial position, the concerted oxidation/reduction cannot occur and the release of a copper atom to bind again in coplanar position, enabling the oxidation/reduction or release of the *o*-diphenol from the active site to the medium. In the case of deoxyarbutin, the *o*-diphenol formed is repulsed by the water due to its hydrophobicity, and so can bind correctly and be oxidized to a quinone before being released. Deoxyarbutin has been characterized with: $k_{\mathrm{cat}}^{D-\mathrm{Arb}}$ = 1.95 ± 0.06 s^-1^ and $K_{M}^{D-\mathrm{Arb}}$ = 33 ± 4 μM. Computational simulations of the interaction of β-arbutin, deoxyarbutin and their *o*-diphenol products with tyrosinase show how these ligands bind at the copper centre of tyrosinase. The presence of an energy barrier in the release of the *o*-diphenol product of deoxyarbutin, which is not present in the case of β-arbutin, together with the differences in polarity and, consequently differences in their interaction with water help understand the differences in the kinetic behaviour of both compounds. Therefore, it is proposed that the release of the *o*-diphenol product of deoxyarbutin from the active site might be slower than in the case of β-arbutin, contributing to its oxidation to a quinone before being released from the protein into the water phase.

## Introduction

Tyrosinase (EC 1.14.18.1) uses molecular oxygen as cosubstrate to catalyse the *ortho*-hydroxylation of monophenols to *o*-diphenols (monophenolase activity), and the oxidation of *o*-diphenols to *o*-quinones (diphenolase activity). The catalytic centre of tyrosinase has two copper atoms each coordinated with three histidine residues, similar to of hemocyanin and catechol oxidase [1,2]. These copper atoms have different oxidation and coordination modes, depending on the enzymatic form: Cu^2+^Cu^2+^ in *E*_m_ (metatyrosinase); Cu^1+^Cu^1+^ in *E*_d_ (deoxytyrosinase); Cu^2+^Cu^2+^$O_{2}^{2-}$ in *E*_ox_ (oxytyrosinase) [2].

This enzyme is responsible for the browning of fruits, vegetables, fungi and crustaceans and is essential in the melanogenesis process of human skin pigmentation for protection from UV-induced damage. Nevertheless, its excessive accumulation can produce hyperpigmentation disorders such as freckles, solar lentigines, ephelide, and melasma [3–5], which have led to the development of mechanisms for inhibition of the enzyme [6–10]. Among these compounds are oxyresveratrol [11], 4-hexylresorcinol [12], 4-*n*-butylresorcinol [13] and ellagic acid [14], which are used in the cosmetics and pharmaceutical industries, although they have recently been characterized as substrates of the enzyme [15–19]. Tyrosinase acts on them when it becomes *E*_ox_, which can be attained in the presence of: a) a reductant, such as ascorbic acid (AH_2_), to convert *E*_m_ to *E*_d_, which evolves to *E*_ox_ in the presence of oxygen; b) hydrogen peroxide (H_2_O_2_), to transform *E*_m_ into *E*_ox_ directly; c) *o*-diphenol (only necessary in catalytic quantities if there is ascorbic acid to keep the quantity of *o*-diphenol constant in the reaction medium) to produce the conversion of *E*_m_ to *E*_d_, which, with oxygen, becomes *E*_ox_ [20].

As in the case of the compounds mentioned above, hydroquinone (HQ) has been described and characterized as an alternative substrate of tyrosinase [20–22]. It has also been seen to induce ochronosis [23] and is a possibly carcinogenic [24] as is kojic acid [25], another inhibitor of tyrosinase. Recently, α-arbutin ((2R,3S,4S,5R,6R)-2-(hydroxymethyl)-6-(4-hydroxyphenoxy)oxane-3,4,5-triol) and β-arbutin ((2R,3S,4S,5R,6S)-2-(hydroxymethyl)-6-(4-hydroxyphenoxy)oxane-3,4,5-triol) (which is the present in nature), previously described as inhibitors, have also been characterized as substrates of mushroom tyrosinase [26].

In order to develop a potent inhibitor of the enzyme, an HQ-derivate called deoxyarbutin (D-Arb) (4-(oxan-2-yloxy)phenol) was synthesized [27], for which a *K*_i_ 10 times lower than that for HQ and 350 times lower than that for arbutin was obtained. Initial studies on the safety and efficacy of the tyrosinase inhibitor D-Arb compared with HQ, demonstrate that D-Arb is 23 times more potent [28]. Moreover, a study comparing HQ, arbutins and D-Arb demonstrated that D-Arb diminishes the expression of tyrosinase [29].

**Fig 1:**
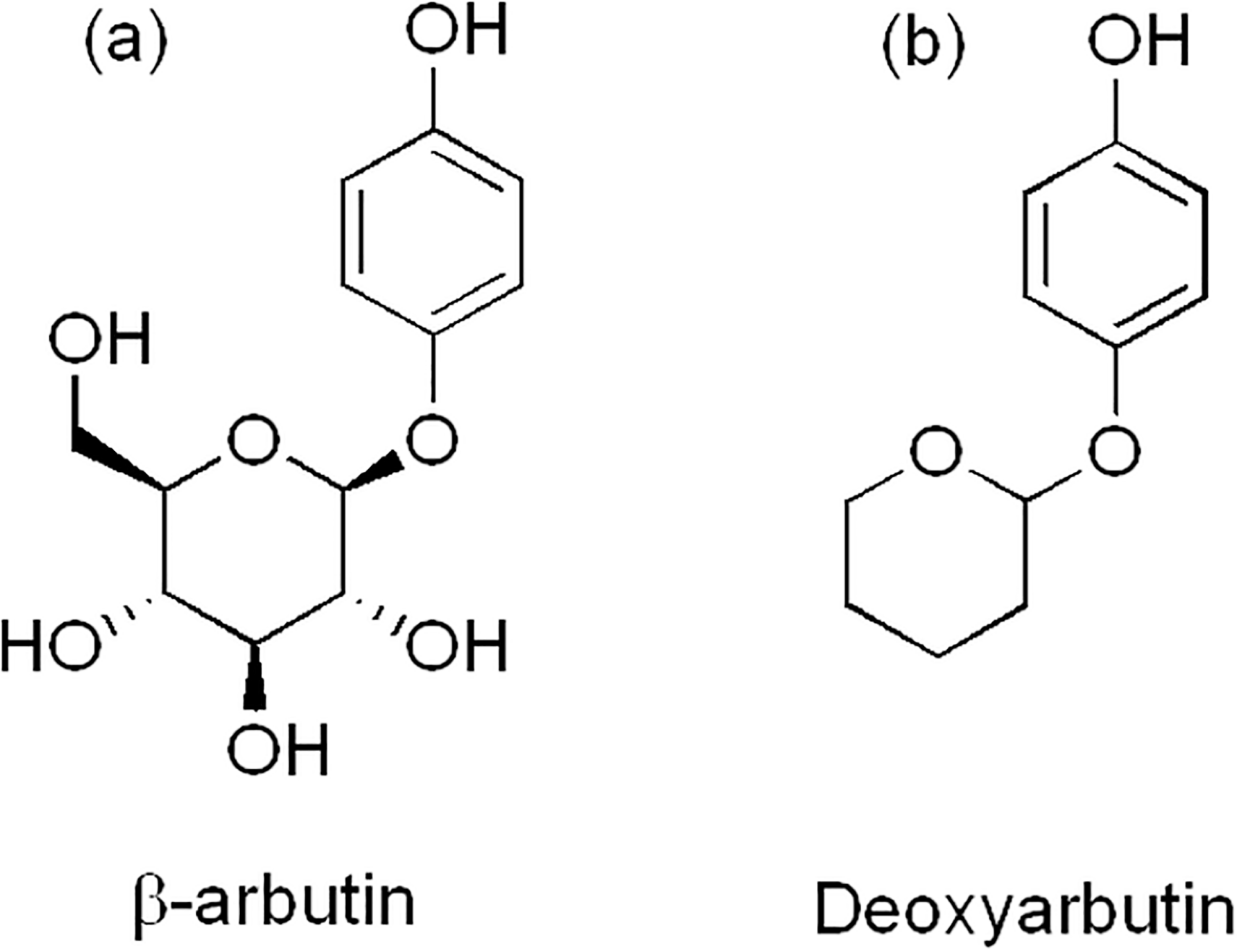
Chemical structures of β-Arb and D-Arb.

For its part, second generation inhibitors of tyrosinase have been synthesized from D-Arb: deoxyfuran (dF), thiodeoxyarbutin (tdA) and flurodeoxyarbutin (fdA), all of which have been demonstrated to be more potent inhibitors than HQ [30] (Fig 1). Regarding the safety of D-Arb compared with arbutins, the Scientific Committee on Consumer Safety (SCCS) has stated that, although the use of D-Arb up to 3% in face creams has been considered safe, the possible formation of HQ make this concentration dangerous [31]. Moreover, the SCCS has stated that the limit in cosmetics should be 2% for face creams and 0.5% in body lotions in the case of α-arbutin [32], and 7% for face creams in the case of β-arbutin (β-Arb) [33].

The toxicity of D-Arb and HQ towards melanosomes, has been studied *in vivo* and *in vitro*, observing that the external membrane of melanosomes was broken in the presence of 5% HQ for 10 days, but not in the presence of D-Arb. Moreover, HQ induced a slightly higher amount of hydroxyl free radicals whereas D-Arb showed moderate hydroxyl radical-scavenging activity, meaning that this latter compound could be used as skin lightening agent and antioxidant with lower cytotoxicity [34].

Derivates of HQ and arbutins, such as D-Arb, could release HQ, which can be toxic for bone marrow due to its catalysis to benzene metabolites [35]. Despite the fact that D-Arb is a more potent inhibitor than HQ and arbutins, its stability under certain conditions remains a problem for use in cosmetics and medicines. It has been demonstrated that D-Arb is a thermolabile and photolabile compound in aqueous solutions [36,37]. Thus, new formulations using anhydrous emulsion systems such as polyol-in-silicone have been developed to stabilize D-Arb [38]. Moreover, nanostructured lipid carriers have been tested to improve the application of D-Arb and hence, its depigmenting ability [39].

The fact that tyrosinase shows activity on D-Arb without the addition of H_2_O_2_, AH_2_ or catalytic amounts of *o*-diphenol encouraged us to see whether it really is a potent inhibitor, as previous works indicated, or an alternative substrate, and to characterize it kinetically.

## Materials and methods

### Materials

Mushroom tyrosinase (3130 U/mg) was supplied by Sigma (Madrid, Spain) and purified as previously described [40]. The protein concentration was determined by Bradford’s method [41], using bovine serum albumin as the standard. The substrates and solvents used were hydrogen peroxide (H_2_O_2_), *tert*-butylcatechol (TBC), L-tyrosine, L-dopa, D-Arb and dimethylformamide (DMF), which were obtained from Sigma (Madrid, Spain). Stock solutions of L-dopa, L-tyrosine, and TBC were prepared in 0.15 mM phosphoric acid to prevent auto-oxidation. Milli-Q system (Millipore Corp, Billerica, MA.) ultrapure water was used throughout. D-Arb was purified to prevent possible contamination by *o*-diphenol by passing it through a column (1 cm diameter) containing 2g of aluminium oxide suspended in 0.5 M ammonium acetate pH 6.1 [42].This solution was further purified by Chelex-100 chromatography (100–200 mesh, Na^+^ form, Biorad) to remove traces of metal ions. Moreover, D-Arb was solubilised in DMF (8%). The chemical structures of β-Arb and D-Arb are shown in Fig 1.

### Spectrophotometric assays

The enzymatic assays were carried out with a PerkinElmer Lambda-35 spectrophotometer, online interfaced with a compatible PC 486DX microcomputer controlled by UV-Winlab software, where the kinetic data were recorded, stored, and analyzed. All of the assays were carried out at 25°C, using 30 mM phosphate buffer at pH 7.25 and the rest of the experimental conditions are specified in the corresponding figure legend [43,44]. Three repetitions of each experiment were made.

### Determination of kinetic parameters

The experimental assays were always carried out in saturating conditions of O_2_ [45–47]. Initial rate values ($V_{0}^{D-\mathrm{Arb}}$), calculated and represented with different concentration of substrate, were fitted to the Michaelis−Menten equation using the Sigma Plot 9.0 program for Windows [48], which provided the maximum rate ($V_{\max}^{D-\mathrm{Arb}}$) and the Michaelis constant ($K_{M}^{D-\mathrm{Arb}}$) in the presence or the absence of inhibitor.

### Action of tyrosinase on D-Arb after adding hydrogen peroxide or ascorbic acid

The action of tyrosinase on D-Arb may occur through the *oxy* form of the enzyme. As the H_2_O_2_ transforms *E*_m_ into *E*_ox_ and the ascorbic acid (AH_2_) reduces *E*_m_ to *E*_d_, which becomes *E*_ox_ in the presence of oxygen, tyrosinase could act on D-Arb in the presence of any of these compounds.

### Determination of ^13^C NMR chemical shifts

^13^C NMR spectra of D-Arb and β-Arb were obtained on a Bruker Avance 300 MHz instrument using DMSO as solvent (S1 Fig). The δ values were measured relative to those for tetramethylsilane using the carbon signals of the deuterated solvent. The maximum line width accepted in the NMR spectra was 0.06 Hz, so that the maximum accepted error for each peak was ± 0.03 ppm.

### Simulation assays

Simulations revealed the kinetic behaviour of the different concentrations of the ligand and enzymatic species involved in the reaction mechanisms proposed for tyrosinase. The respective systems of differential equations were solved numerically for particular sets of values of the rate constants and initial concentrations. Numerical integration is based on the Runge-Kutta-Fehlberg algorithm [49], implemented on a PC-compatible computer program (WES) [50]. Simulations intend to reproduce the qualitative dependences of the integrated mechanism in relation with the experimental data. The kinetic rate constants used in the simulations with L-tyrosine and L-dopa (S18 Fig) agree with previous studies carried out by our group [40,44,51]. In the case of D-Arb, we use $k_{\mathrm{cat}}^{D-\mathrm{Arb}}=k_{10}^{}$ = 1.5 s^-1^, $K_{M}^{D-\mathrm{Arb}}=(k_{10}^{}{+k}_{-9}^{})/k_{9}^{}$ = 0.033 mM, which were obtained along this work, and the rest of constants are estimates to reproduce the qualitative experimental dependences.

### Molecular dynamics (MD)

The molecular structure of tyrosinase was taken from the Protein Databank (PDB ID:2Y9W, Chain A) [52], corresponding to the *deoxy* form of tyrosinase from *Agaricus bisporus*. Information on the chemical structures for β-Arb and D-Arb is available in the PubChem Substance and Compound database [53] through the unique chemical structure identifier CID: 346 for β-Arb and CID: 1745519 for D-Arb.

MD simulations were conducted using GROMACS, version 5.0.7 [54]. The protein was oriented with the z-axis running from the copper ions to the outside of the protein structure along the ligand entry/exit path. To parameterize the protein and ions the 53A6 GROMOS force field [55] included in GROMACS package was used. Ligands were parameterized with the same force field using the Automated Topology Builder server [56] version 2.2 using Quantum Mechanical optimization in water at the B3LYP/6-31G* level. The hydration free energy in SPC water calculation was validated by the ATB server for the GROMOS force field by using a set of 459 molecules of known experimental values [56]. To further refine the ligand topologies a more extensive validation would be required.

The initial configuration was constructed by placing the ligands in the active site of the protein and the system was solvated with water from a simple point charge (SPC) water model [57] at a density of 33.5 water molecules/nm^3^ in a box of 7.2x7.2x7.2 nm^3^ size. After a steepest-descent minimization step the system was equilibrated in an NpT ensemble for 2 ns with a time step of 2 fs, using a V-rescale [58] and Berendsen barostat [59] to regulate temperature and pressure respectively. Position restraints were applied on the protein including the copper ions. Production run were performed in the same conditions with a Parrinello-Rahman barostat for 1 ns.

### Potential of mean force

The *ortho*-hydroxy derivatives of β-Arb and D-Arb were constructed with PyMOL 1.8.2.1 [60]. MD simulations were carried out as described above for arbutin and D-Arb to obtain the equilibrated structures of the hydroxy-ligands in the active site. To calculate potentials of mean force (PMF) the final configuration of the production run was used as the initial structure for the “pulling” simulation. The centre of mass distances (COM) between the carbon atom at *para* position of the aromatic ring of each ligand and the centre of mass of the copper ions were calculated, and then pulled away from the copper site along the z-axis in a time of 500 picoseconds (ps) using a spring constant of 3000 kJ^-1^ nm^-2^ and a pulling rate of 0.0034 nm ps^-1^. This pulling was carried out to create a set of starting configurations. The values of the spring constant and pulling rate were chosen after testing many combinations to find an optimal pair of numbers that did not cause instabilities in the system. A total of 40 windows were used distributed within a 1.7 nm COM separation. In each window, a short equilibration of 1 ns was followed by a 5 ns production run for umbrella sampling. A harmonic force with a force constant of 3000 kJ mol^-1^ nm^-2^ was applied for each umbrella-sampling window. Additional windows were generated to improve sampling. Thus, a total of 160 and 254 windows were finally used for β-ArbOH and D-ArbOH, respectively. To generate the potentials of mean force, the weighed histogram analysis method (WHAM) was used [61].

## Results

### D-Arb apparently inhibits the monophenolase and diphenolase activities of tyrosinase

**Fig 2:**
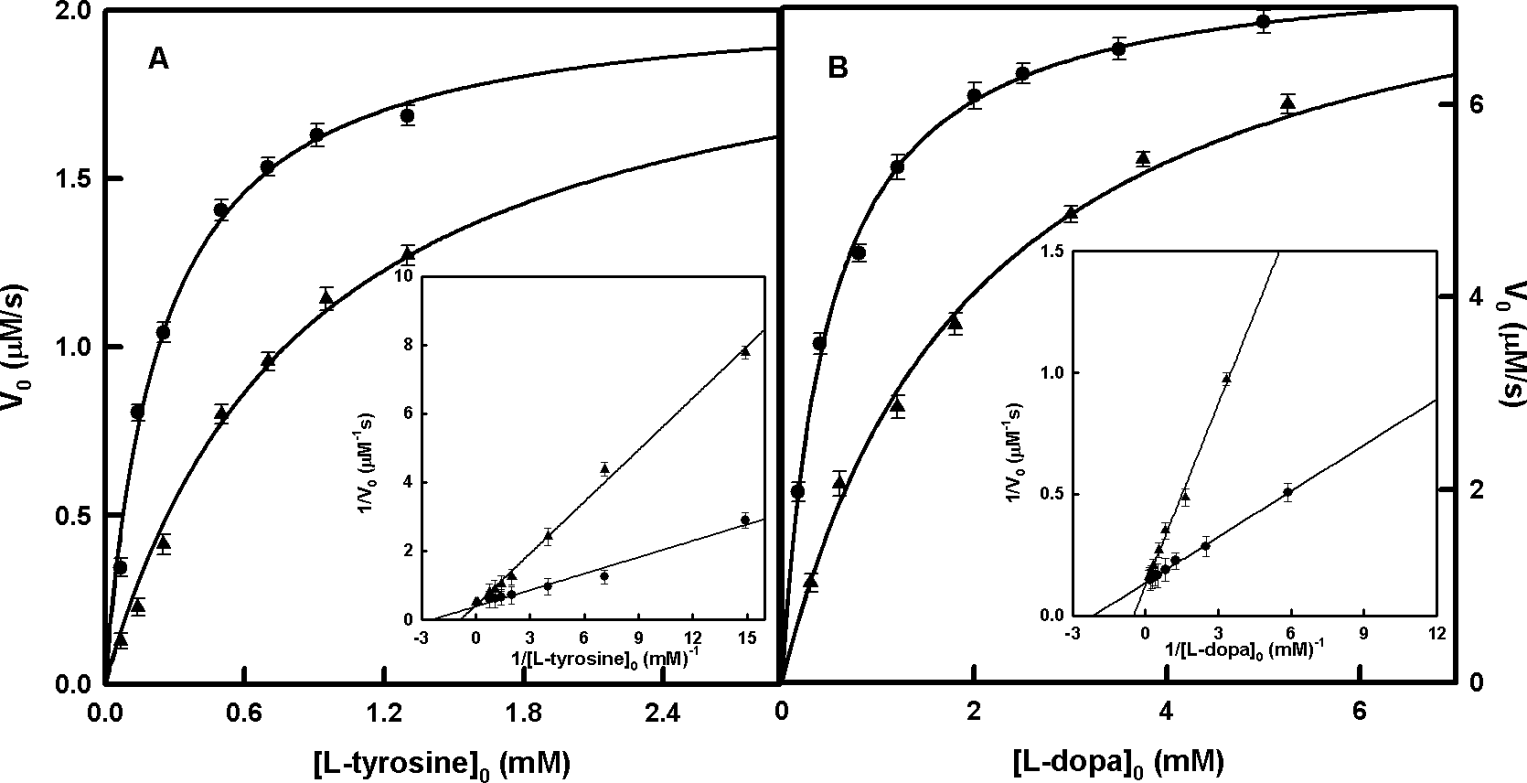
Apparent inhibition of tyrosinase by D-Arb. **A.** Representation of initial rate values of tyrosinase on L-tyrosine in the absence (●) and presence (▲) of D-Arb (0.2 mM). The increase of absorbance corresponding to the apparent formation of dopachrome was followed at 475 nm. The experimental conditions were [*E*]_0_ = 90 nM and R = [L-dopa]_0_ / [L-tyrosine]_0_ = 0.042. **Inset.** Graphical representation of the Lineweaver–Burk equation to show the inhibition of the monophenolase activity of tyrosinase in the absence (●) and presence (▲) of D-Arb (0.2 mM). The experimental conditions were the same as in the main figure. **B.** Representation of initial rate values of tyrosinase on L-dopa in the absence (●) and presence (▲) of D-Arb (0.2 mM). The experimental conditions were [*E*]_0_ = 60 nM. **Inset.** Graphical representation of the Lineweaver–Burk equation showing the inhibition of the diphenolase activity of tyrosinase in the absence (●) and presence (▲) of D-Arb (0.2 mM). The experimental conditions were the same as in the main figure.

When these activities of the enzyme on L-tyrosine and L-dopa are studied in the presence of D-Arb, apparent inhibition is observed (Fig 2A and 2B). When the type of inhibition was studied (Fig 2A Inset and 2B Inset), the results showed an apparent competitive inhibition. The $K_{i}^{\mathrm{app}}$ values are shown in Table 1.

**Table 1:** Type and kinetic constants for the apparent inhibition of tyrosinase by D-Arb and β-Arb.

Compound	$K_{I}^{\mathrm{app}}$ (mM)		Type		Reference
	Monophenolase	Diphenolase	Monophenolase	Diphenolase	
**D-Arb**	0.078 ± 0.005	0.040 ± 0.002	Competitive	Competitive	This paper
**β-Arb**	1.42 ± 0.08	0.9 ± 0.05	Competitive	Competitive	[26]

### Total oxygen consumption test

**Fig 3:**
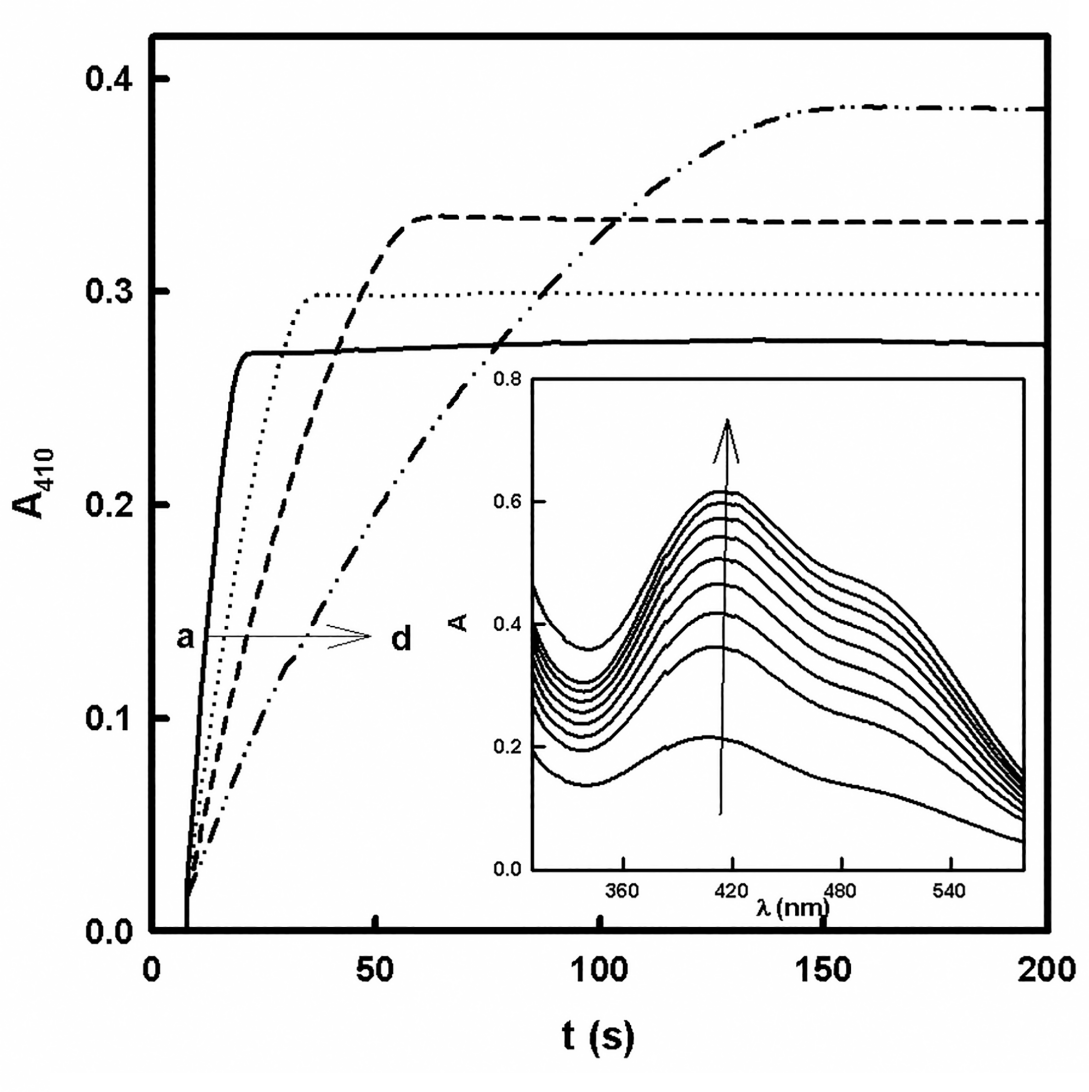
Action of tyrosinase on TBC in the presence of D-Arb. The increase of absorbance was followed at 410 nm by means of a total oxygen consumption test, carried out in the presence of TBC and different concentrations of D-Arb (mM): a) 0, b) 0.1, c) 0.2 and d) 0.4. The rest of the experimental conditions were [*E*]_0_ = 50 nM and [TBC]_0_ = 1 mM. **Inset.** Spectrophotometric recordings of the action of tyrosinase on TBC and D-Arb. The experimental conditions were [*E*]_0_ = 20 nM, [TBC]_0_ = 0.5 mM and [D-Arb]_0_ = 0.4 mM. The spectrophotometric recordings were made every 60 seconds.

In previous works [18,26], we developed a test to elucidate whether a compound is a substrate or inhibitor of tyrosinase. The method consists of a spectrophotometric measurement at 410 nm of the formation of the *o*-*tert*-butylquinone generated by the action of tyrosinase on TBC in the absence (recording “a”) and presence of increasing concentrations of D-Arb (recordings “b-d”) (Fig 3). The absorbance at 410 nm and the reaction time increase as the concentration of D-Arb increases, which indicates that a product with a higher absorbance than that of *o*-*tert*-butylquinone comes from D-Arb. Moreover, the formation of a new product is detected in the spectrophotometric recordings of the action of tyrosinase in a medium with TBC and D-Arb (Fig 3 Inset).

### Action of tyrosinase on D-Arb after adding hydrogen peroxide or ascorbic acid

*E*_m_ becomes *E*_ox_ in the presence of H_2_O_2_ [62], and it was expected that this form of tyrosinase would be able to hydroxylate D-Arb. The result of the action of tyrosinase in the presence of H_2_O_2_ is shown in S2 Fig and the effect of the variation of the concentration of enzyme in S2 Fig Inset.

For its part, AH_2_ is a reductant able to reduce *E*_m_ (Cu^2+^Cu^2+^) to *E*_d_ (Cu^1+^Cu^1+^), which in the presence of oxygen becomes *E*_ox_ (Cu^2+^Cu^2+^$O_{2}^{2-}$) [20], which is active on D-Arb (S3 Fig), as confirmed above. The activity observed is due to the quinone generated, but as the amount of ascorbic acid is small, it cannot reduce the quinone [20].

### Action of tyrosinase on D-Arb without the addition of hydrogen peroxide or ascorbic acid

**Fig 4:**
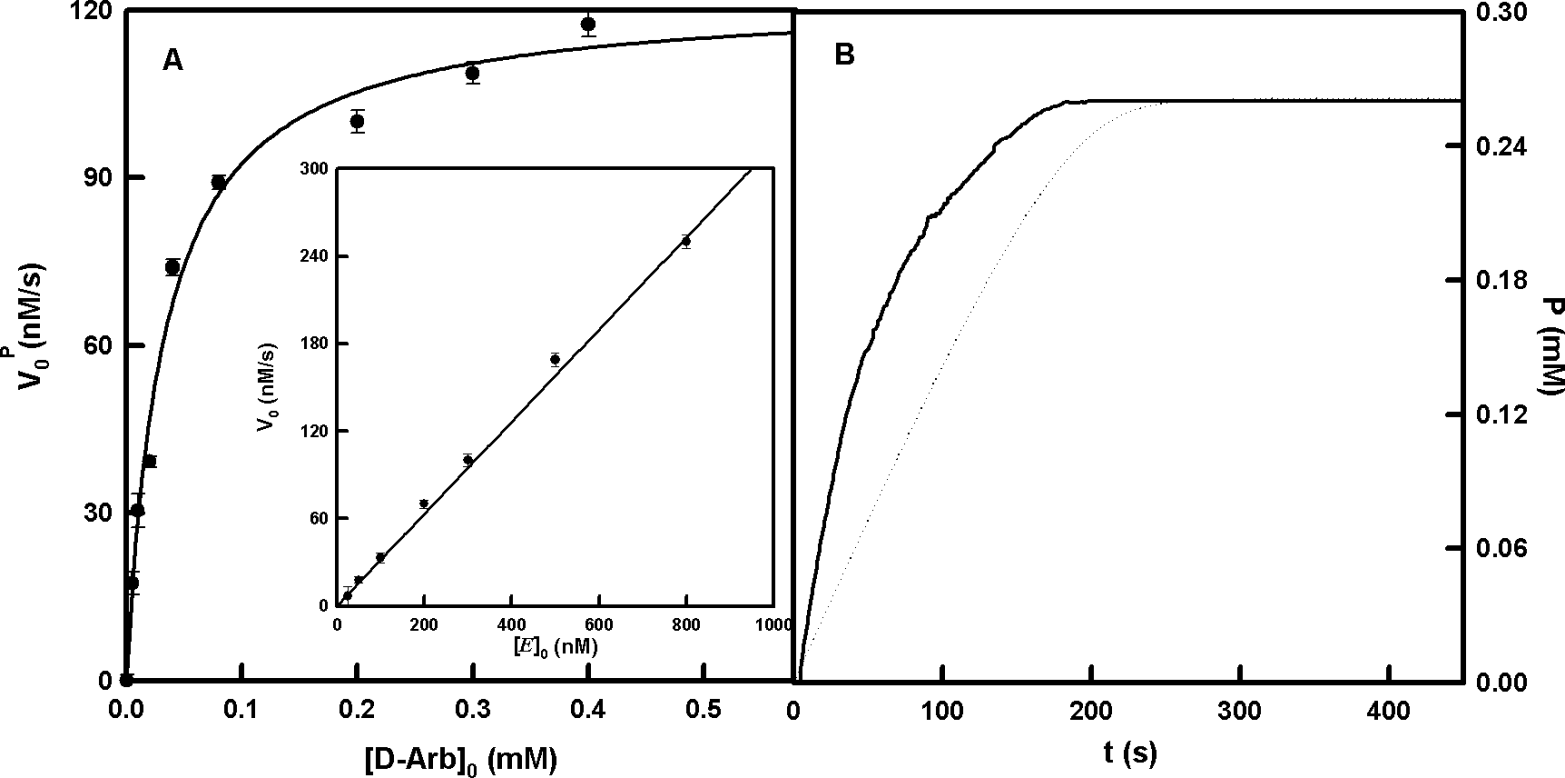
Action of tyrosinase on D-Arb without the addition of H_2_O_2_ or AH_2_. **A.** Representation of the initial formation rate of quinone (P) due to the action of tyrosinase on different concentrations of D-Arb. The rest of the experimental conditions were: [*E*]_0_ = 300 nM. **Inset.** Representation of the initial formation rate of quinone (P) due to the action of tyrosinase on D-Arb (0.2 mM) with different concentrations of enzyme. **B.** Action of tyrosinase on D-Arb until total consumption of the oxygen (—). The spectrophotometric recording was made at λ = 485 nm, and the concentration of quinone (P) was calculated considering the molar absorptivity value (ε) as 2300 M^-1^ cm^-1^. The experimental conditions were [*E*]_0_ = 1.3 μM and [D-Arb]_0_ = 0.4 mM. Formation of quinone (P) during the simulation of the mechanism of Fig 5 until total consumption of the oxygen (…). The simulated conditions were [*E*]_0_ = 1.5 μM, [*E*_ox_]_0_ = 0.2 x [*E*]_0_, [*E*_m_]_0_ = 0.8 x [*E*]_0_; [H_2_O_2_]_0_ = 1.25 μM, [D-Arb]_0_ = 0.4 mM and [O_2_]_0_ = 0.26 mM. The rate constants were: *k*_8_ = 0.5 x 10^7^ M^-1^ s^-1^, *k*_-8_ = 5 x 10^3^ s^-1^, *k*_9_ = 1.6 x 10^5^ M^-1^ s^-1^, *k*_-9_ = 3.8 s^-1^, *k*_10_ = 1.5 s^-1^, *k*_11_ = 400 s^-1^, *k*_12_ = 1.6 x 10^5^ M^-1^ s^-1^, *k*_-12_ = 3.8 s^-1^, *k*_15_ = 2 x 10^6^ M^-1^ s^-1^, *k*_-15_ = 10 s^-1^.

**Fig 5:**
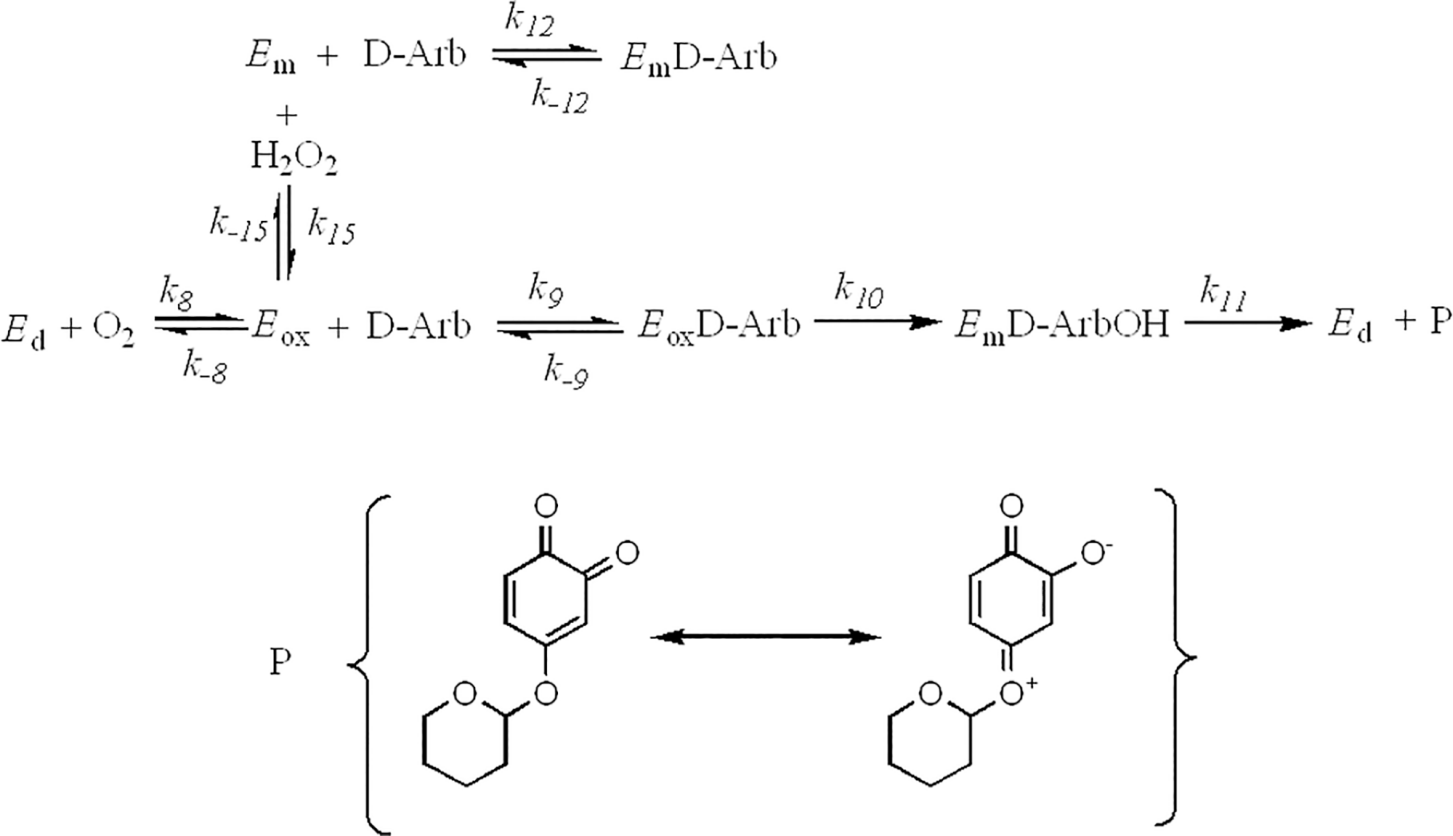
Schematic representation of the action mechanism of tyrosinase on D-Arb. D-Arb = deoxyarbutin, D-ArbOH = hydroxylated deoxyarbutin, P = quinone derived from hydroxylated deoxyarbutin, *E*_m_ = metatyrosinase, *E*_d_ = deoxytyrosinase and *E*_ox_ = oxytyrosinase.

The experiments represented in Fig 4A show that tyrosinase acts on D-Arb without any reductant or H_2_O_2_, behaviour which has not been observed previously in similar compounds (monophenols). *E*_ox_ is the only form of tyrosinase which can hydroxylate D-Arb, but the cycle of *E*_ox_ has to be completed to maintain the activity (Fig 5). According to the experimental and simulated results shown in Fig 4B, the stoichiometry of product (P) formation with respect to the oxygen consumed would be 1:1.

The initial formation rate of P is given by the equation:

$$V_{0}^{P}=\frac{\alpha_{1}{\left[D-\mathrm{Arb}\right]}_{0}{\left[O_{2}\right]}_{0}{\left[H_{2}O_{2}\right]}_{0}^{}}{\begin{array}{l} \beta_{1}{\left[H_{2}O_{2}\right]}_{0}+\beta_{2}{\left[O_{2}\right]}_{0}+\beta_{3}{\left[H_{2}O_{2}\right]}_{0}{\left[D-\mathrm{Arb}\right]}_{0}+\beta_{4}{\left[D-\mathrm{Arb}\right]}_{0}{\left[O_{2}\right]}_{0}+\\ \beta_{5}{\left[H_{2}O_{2}\right]}_{0}{\left[O_{2}\right]}_{0}+\beta_{6}{\left[H_{2}O_{2}\right]}_{0}{\left[O_{2}\right]}_{0}{\left[D-\mathrm{Arb}\right]}_{0} \end{array}}$$ (1)

$\alpha_{1}=k_{8}\;k_{9}\;k_{10}\;k_{11}\;k_{12}\;k_{15}$
$\beta_{1}=k_{-8}\;k_{11}\;k_{12}\;k_{15}\;(k_{-9}+\;k_{10})$
$\beta_{2}=k_{8}\;k_{11}\;k_{12}\;k_{-15}\;(k_{-9}+k_{10})$
$\beta_{3}=k_{9}\;k_{10}\;k_{11}\;k_{12}\;k_{15}$
$\beta_{4}=k_{8}\;k_{11}\;k_{-15}\;(k_{-9}+k_{10})$
$\beta_{5}=k_{8}\;k_{11}\;k_{12\;}\;k_{15}\;(k_{-9}+k_{10})$
$\beta_{6}=k_{8}\;k_{9}\;k_{12\;}\;k_{15}\;(k_{10}+k_{11})$

As the enzyme is saturated by oxygen ([O_2_]_0_ → ∞) [45–47], we obtain:

$$V_{0}^{P}=\frac{\frac{k_{10}k_{11}}{k_{10}{+k}_{11}}{\left[D-\mathrm{Arb}\right]}_{0}{\left[H_{2}O_{2}\right]}_{0}^{}}{\begin{array}{l} \frac{k_{11}k_{-15}(k_{-9}{+k}_{10})}{k_{9}k_{15}(k_{10}+k_{11})}+\frac{k_{11}k_{-15}(k_{-9}{+k}_{10})}{k_{9}k_{12}k_{15}(k_{10}{+k}_{11})}{\left[D-\mathrm{Arb}\right]}_{0}+\\ \frac{k_{11}(k_{-9}{+k}_{10})}{k_{9}(k_{10}{+k}_{11})}{\left[H_{2}O_{2}\right]}_{0}+{\left[H_{2}O_{2}\right]}_{0}{\left[D-\mathrm{Arb}\right]}_{0} \end{array}}$$ (2)

as *k*_11_ >> *k*_10_, so:

$$V_{0}^{P}=\frac{k_{10}{\left[D-\mathrm{Arb}\right]}_{0}{\left[H_{2}O_{2}\right]}_{0}^{}}{\begin{array}{l} K_{15}\frac{k_{-9}{+k}_{10}}{k_{9}}+\frac{K_{15}}{K_{12}}\left(\frac{k_{-9}{+k}_{10}}{k_{9}}\right){\left[D-\mathrm{Arb}\right]}_{0}+\\ \frac{k_{-9}{+k}_{10}}{k_{9}}{\left[H_{2}O_{2}\right]}_{0}+{\left[H_{2}O_{2}\right]}_{0}{\left[D-\mathrm{Arb}\right]}_{0} \end{array}}$$ (3)

being

$$K_{M}^{D-\mathrm{Arb}}=\frac{k_{-9}{+k}_{10}}{k_{9}}$$

$$K_{15}=\frac{k_{-15}}{k_{15}}$$

$$K_{12}=\frac{k_{-12}}{k_{12}}$$

hence

$$V_{0}^{P}=\frac{k_{10}{\left[D-\mathrm{Arb}\right]}_{0}}{K_{M}^{D-\mathrm{Arb}}\left(\frac{K_{15}}{{\left[H_{2}O_{2}\right]}_{0}}+\frac{K_{15}{\left[D-\mathrm{Arb}\right]}_{0}}{K_{12}{\left[H_{2}O_{2}\right]}_{0}}+1\right)+{\left[D-\mathrm{Arb}\right]}_{0}}$$ (4)

so

$$K_{15}=\frac{k_{-15}}{k_{15}}=\frac{{\left[E_{m}\right]}_{0}{\left[H_{2}O_{2}\right]}_{0}}{{\left[E_{\mathrm{ox}}\right]}_{0}}=R^{E}{\left[H_{2}O_{2}\right]}_{0}$$ (5)

being

$$R^{E}=\frac{{\left[E_{m}\right]}_{0}}{{\left[E_{\mathrm{ox}}\right]}_{0}}$$

$$V_{0}^{P}=\frac{k_{10}{\left[D-\mathrm{Arb}\right]}_{0}{\left[E\right]}_{0}}{K_{M}^{D-\mathrm{Arb}}\left(R^{E}+R^{E}\frac{{\left[D-\mathrm{Arb}\right]}_{0}}{K_{12}}+1\right)+{\left[D-\mathrm{Arb}\right]}_{0}}=\frac{\frac{k_{10}}{1+\frac{K_{M}^{D-\mathrm{Arb}}}{K_{12}}R^{E}}{\left[D-\mathrm{Arb}\right]}_{0}{\left[E\right]}_{0}}{\frac{K_{M}^{D-\mathrm{Arb}}\left(1+R^{E}\right)}{1+\frac{K_{M}^{D-\mathrm{Arb}}}{K_{12}}R^{E}}+{\left[D-\mathrm{Arb}\right]}_{0}}$$ (6)

As $K_{M}^{D-\mathrm{Arb}}=\left(k_{-9}{+k}_{10}\right){/k}_{9}$ and *K*_12_ = *k*_−12_/*k*_12_, if we consider that *K*_9_ = *k*_−9_/*k*_9_ could have a similar value to *K*_12_ (since they refer to the bond to the same substrate of two different forms of the enzyme, oxy and metatyrosinase, but the same active site), $K_{M}^{D-\mathrm{Arb}}$ would be similar to *K*_12_, since *k*_10_ has a low value. If this constant has a high value, $K_{M}^{D-\mathrm{Arb}}$ would be higher than *K*_12_, as it happens in the case of true substrates of tyrosinase [63,64].

Therefore,

if $K_{M}^{D-\mathrm{Arb}}\approx K_{12}$

$$V_{0}^{P}=\frac{\frac{k_{10}}{1+R^{E}}{\left[D-\mathrm{Arb}\right]}_{0}{\left[E\right]}_{0}}{\frac{K_{M}^{D-\mathrm{Arb}}\left(1+R^{E}\right)}{1+R^{E}}+{\left[D-\mathrm{Arb}\right]}_{0}}=\frac{k_{10}{\left[D-\mathrm{Arb}\right]}_{0}{\left[E_{\mathrm{ox}}\right]}_{0}}{K_{M}^{D-\mathrm{Arb}}+{\left[D-\mathrm{Arb}\right]}_{0}}$$ (7)

where

$${\left[E_{ox}\right]}_{0}=\frac{{\left[E\right]}_{0}}{1+R^{E}}=\frac{{\left[E\right]}_{0}}{5}=0.2{\left[E\right]}_{0}$$ (8)

The following characteristics of the action of the enzyme on D-Arb should be noted:

There is no lag period because the product (P) is a stable quinone (Fig 4A). Moreover, D-Arb was purified by passing it through a column containing aluminium oxide in order to eliminate possible contamination with *o*-diphenol. S4 Fig shows the spectra before (a) and after (b) purification in the column, the oxidation by sodium periodate (the absorbance would change in the presence of *o*-diphenol) (S4 Fig Inset A) and the activity of tyrosinase on the eluted D-Arb with no lag (S4 Fig Inset B). The absence of variation in the absorbance after the oxidation with sodium periodate and the absence of the lag period in the activity of tyrosinase on the eluted D-Arb demonstrate that there was no contamination by *o*-diphenol.

### Kinetic characterization of D-Arb as substrate of tyrosinase

The experiments mentioned above demonstrate that *E*_ox_ is able to act on D-Arb and, so, the proposed mechanism in Fig 5 is valid. The kinetic characterization of the action of tyrosinase on D-Arb should be based on a measurable product (P).

### Formation and properties of the product of the enzymatic reaction (P)

The enzyme hydroxylates D-Arb to D-ArbOH in a step controlled by *k*_10_, and subsequently oxidizes this compound to a quinone (P), in which the *p*-quinoid canonical form contributes significantly to the hybrid resonance, in a stage controlled by *k*_11_. The substrate is consumed by the enzymatic action (S5 Fig Inset), which allows the molar absorptivity value (ε) to be determined (S5 Fig). The action of tyrosinase on different concentrations of D-Arb was tested to calculate the $V_{0}^{D-\mathrm{Arb}}$, the values of which were fitted by non-linear regression to equation 7, thus providing the $k_{\mathrm{cat}}^{D-\mathrm{Arb}}$ and $K_{M}^{D-\mathrm{Arb}}$ values (Table 2). Note the low value of $k_{\mathrm{cat}}^{D-\mathrm{Arb}}$, which agrees with the chemical shift values of the carbon with the phenolic hydroxyl group (Table 2). The low value of $K_{M}^{D-\mathrm{Arb}}$ (Table 2) also agrees with the low value of $k_{\mathrm{cat}}^{D-\mathrm{Arb}}$ and the high value of *k*_9_, which reflects the hydrophobicity of D-Arb. The differences with β-Arb (Table 2) is that this compound have many hydroxyl groups, which interact with residues of the active site of the enzyme.

**Table 2:** Kinetic constants for the characterization of the activity of tyrosinase on D-Arb and β-Arb and chemical shift values of carbon with the phenolic hydroxyl group.

Compound	*k*_cat_ (s^-1^)	*K*_M_ (mM)	δ_1_ (ppm)	Reference
**D-Arb**	1.95 ± 0.06	0.033 ± 0.004	150.12	This paper
**β-Arb**	3.77 ± 0.29	3 ± 0.19	151.22	[26]

### Generation and disappearance of the *oxy* form of tyrosinase

**Fig 6:**
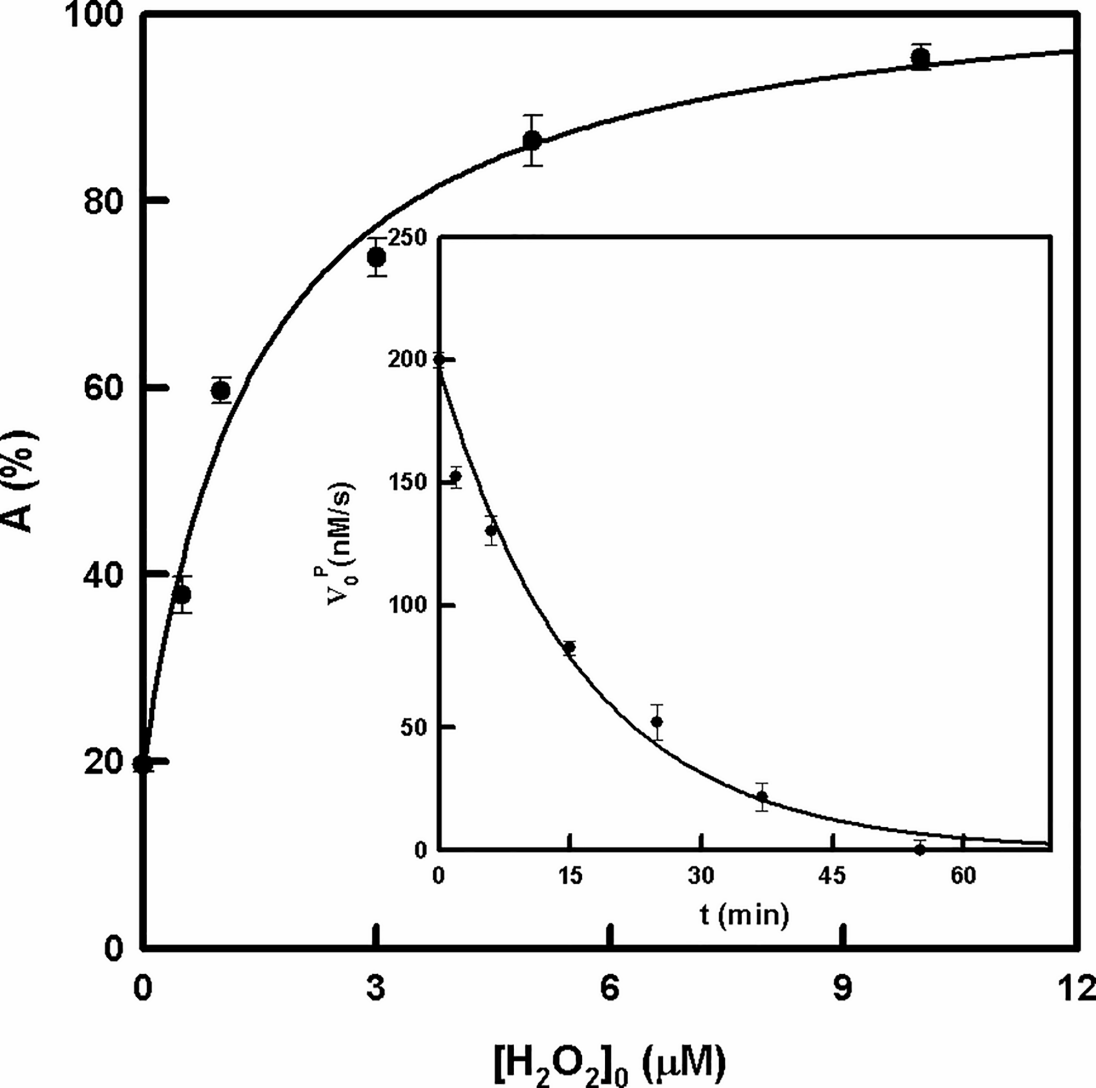
Generation and disappearance of the *oxy* form of tyrosinase. Degree of activation of the activity of tyrosinase on D-Arb (0.2 mM) after 1 minute of pre-incubation with different concentrations of H_2_O_2_. The rest of the experimental conditions were: [*E*]_0_ = 0.1 μM. **Inset.** Degree of inhibition of the activity of tyrosinase on D-Arb (0.4 mM) after different times of pre-incubation with catalase (930 U/ml). The rest of the experimental conditions were: [*E*]_0_ = 0.5 μM.

The system changes when a micromolar range of H_2_O_2_ is added (Fig 5). The reason for this is that H_2_O_2_ increases the concentration of *E*_ox_ and so the activity is higher (Fig 6) [65]. On the other hand, the addition of catalase leads to the consumption of H_2_O_2_, shifting the equilibrium toward the right, decreasing the concentration of *E*_ox_ and hence, the enzymatic activity, as demonstrated in Fig 6 Inset [65]. Simulation of the mechanism proposed in Fig 5, adding the action of catalase described above, provides the same result (S6 Fig). A similar situation is observed when the mechanism of Fig 5 is simulated adding the possibility of D-ArbOH being released to the medium. In this case, the mechanism was simulated by means of two alternatives: a) oxidizing D-ArbOH by oxygen immediately (as represented in S7 Fig) in S8B Fig) allowing *E*_m_ and *E*_ox_ to bind to D-ArbOH, giving rise to the complex *E*_ox_D-ArbOH, which leads to the formation of *E*_m_ + P (as represented in S9 Fig) in S10 Fig It can be seen that the product formation process in S8 Fig stops because the *E*_ox_ is consumed, while S10 Fig tends to the same situation, but needs more time. In both cases, the activity stops because all the enzyme becomes *E*_m_, which is not active on D-Arb [42]. These figures differ from S11 Fig, in which the formation of product according to Fig 5 is simulated, obtaining a straight line when the same time as that shown in S8 and S10 Figs is used. This indicates that the system does not stop, as was observed experimentally, which means that D-ArbOH is not released.

### Computational simulations of substrates binding

The chemical structures of β-Arb and D-Arb (Fig 1) suggest that the compounds have a very different behaviour with respect to environmental polarity. β-Arb contains four hydroxyl groups, D-Arb lacks, so that the partition coefficient (log P) is -1.35 and +2.40 for β-Arb and D-Arb, respectively, as calculated by the XLogP3 program [66]. These values indicate that β-Arb is preferably partitioned in water while D-Arb is partitioned in octanol. Therefore, polar interactions, like hydrogen bonds with the proteins and the water molecule of the solvent, are very important for defining the ligand binding interactions that take place in the substrate binding pocket. The *oxy* form of tyrosinase was solvated with water in a rectangular box. MD simulation was carried out to equilibrate the system in water solvent with the ligand bound to the copper centre.

**Fig 7:**
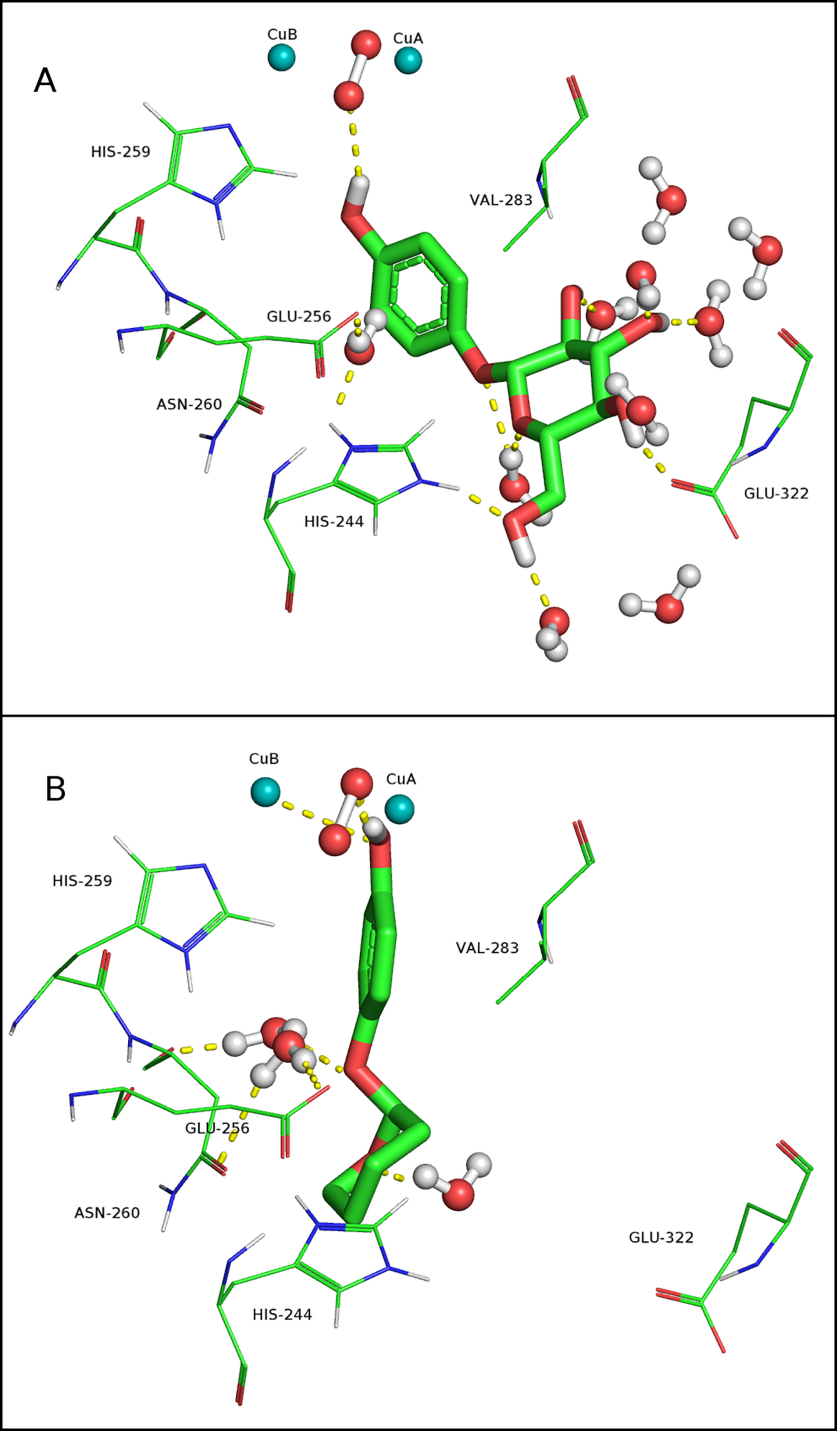
Computational results for the *oxy* form of mushroom tyrosinase (*Agaricus bisporus*). A representative MD snapshot is shown for the configurations of β-arbutin (A) and deoxyarbutin (B) in the active centre. The atom colours are as follows: red = oxygen, blue = nitrogen, light blue (spheres) = copper, green = carbon and white = hydrogen. In yellow dashed lines: possible hydrogen bonds interactions. Only the most relevant residues are depicted.

Fig 7 shows a representative MD snapshot of the configurations of β-Arb and D-Arb in the active centre of oxytyrosinase. Their phenolic groups interact with the oxygen molecule by hydrogen bonds and by electrostatic interaction with a copper atom. However, both ligands show a very different configuration in the docking pose at the active site. Only water molecules in a radius of 4 Å from the ligand molecule are depicted to exclude water molecules that are outside the interaction distance. Of note is the difference in the amount of water around both ligands. β-Arb is surrounded by many more water molecules than D-Arb, as might be expected from their polarity differences.

Another view of Fig 7 is shown in S12 Fig to highlight the position of the oxane ring of D-Arb, which is more restricted to the cavity of the active site, while the pyranose ring of β-Arb is more exposed to the outermost part of the active site. It is therefore plausible that the amount of water in the active site might be an important factor to take into consideration to modulate the affinity of ligands. Usually, water molecules are removed from the protein in docking studies. However, some structurally conserved water molecules participating in substrate binding are found in the active site of the crystallized tyrosinase, and have been proposed as being involved in substrate binding and catalysis [67–69]. All these results reveal the importance of water molecules in the process of binding to the active site.

### Computational simulations of *o*-diphenol binding

This study has demonstrated that the *o*-diphenol product of D-Arb is not released from the enzyme but is oxidized to the corresponding quinone, while other substrates, like β-Arb, are hydroxylated by oxytyrosinase, releasing *o*-diphenol. This result suggests that the *o*-diphenol product of D-Arb is somehow trapped in the active site long enough to be oxidized before being released from the enzyme. In a search for possible reasons to explain this behaviour the *o*-diphenol products of D-Arb (D-ArbOH) and β-Arb (β-ArbOH) were studied by MD in the *met* form of tyrosinase.

The distribution of water molecules along the z-axis from copper atoms to the outside of the cavity of the substrate binding pocket of metatyrosinase was determined in the presence of bound *o*-diphenols. For this purpose, the quantity of water molecules in a cylinder of 0.6 nm radius centred at the *o*-diphenol molecule was determined.

S13 Fig shows the histograms of the distribution of the water molecules. β-ArbOH, bound to the copper atoms goes towards the water layer, the pyranose ring being surrounded by water molecules (S13A Fig). In contrast, D-ArbOH interacts with water almost exclusively trough ether oxygen atoms. The oxane ring excludes water molecules due to its high hydrophobicity and the oxane ring is rotated with respect to the phenyl ring increasing the distance from the water layer and avoiding the interaction with water in the outer region of the binding pocket (S13B Fig).

It is feasible to think that D-ArbOH has an energetic barrier formed by the water layer, which would slow down its release from the cavity, contributing to keeping D-ArbOH longer in the active centre and facilitating its oxidation to quinone.

**Fig 8:**
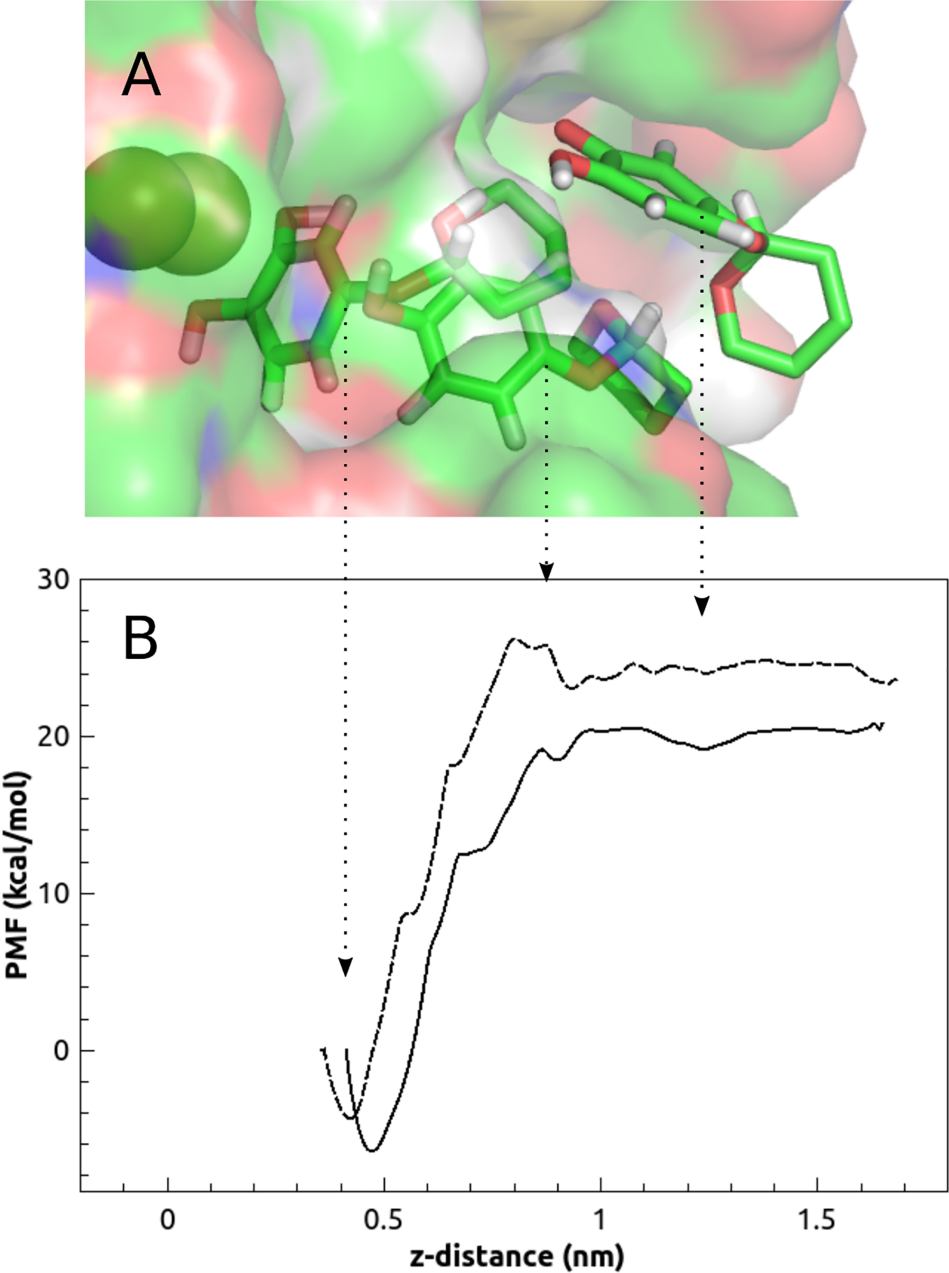
Potential of mean force (PMF) as a function of the z-distance between the carbon atoms at *para* position of *o*-diphenols. (A) Surface representation of the localization of ligand in the binding cavity of metatyrosinase. (B) Potential of mean force curves for β-ArbOH (solid line) and D-ArbOH (dashed line).

Potential of mean force (PMF) profiles for the dissociation process of *o*-diphenols from metatyrosinase along the z-axis are presented in Fig 8. The greatest z-distance values correspond to the *o*-diphenols in the bulk solution. One of the differences between the two *o*-diphenols that deserves to be highlighted is that the PMF profile of D-ArbOH shows an energy barrier of about 3 kcal/mol at a distance of 0.8 nm, which is not observed in the β-ArbOH PMF profile. Attempts to calculate the free energy of the *o*-diphenol dissociation provide very high values. However, it has to be taken into account that the potential of mean force includes the contribution of all the components of the system. Since the protein is computed in MD under position restraint, the main uncertainty arises from the water molecules which have freedom of movement and change their electrostatic interactions.

To throw light on the events occurring in the energy barrier of the PMF profile of D-ArbOH (0.7–1 nm of Fig 8B) the most relevant hydrogen bond interactions were determined for both *o*-diphenols using GROMACS tools (S14 Fig). One important thing to mention is that the amount of water bound to β-ArbOH is much higher all along the trajectory compared with D-ArbOH (S14 Fig). β-ArbOH forms a total of 9 hydrogen bonds at the active site (z = 0.42 nm), decreases to 8 and finally reaches 12 in the bulk solution (z > 1.1 nm). However, D-ArbOH forms about 3 hydrogen bonds at the active site, decreases to 1–2 above 0.6–0.75 nm z-distance and increases to 5 in the bulk solution. This means that the pyranose ring of β-ArbOH is always bound to water, as was described for β-Arb binding to oxytyrosinase (Fig 7A). The hydrogen bonds between all the oxygen atoms of D-ArbOH and different aminoacid residues and water were measured. Among then, only significant differences were found in the 0-7-1 nm z-distance between the phenolic groups of D-ArbOH and water molecules (S14B Fig) and N260 (S14C Fig). It can be seen that the hydroxyl group of C2 (C_2_-OH) forms 2 hydrogen bonds at the active site (0.41 nm) while that of hydroxyl group at C1 (C_1_-OH) does not (S14B Fig). Between 0.6 and 0.72 nm, the loss of hydrogen bonds appears to be concomitant with the formation of one hydrogen bond with N260 and vice versa (S14C Fig). Afterwards, C_1_-OH displaces C_2_-OH from the N260 interaction (0.76 nm) and at 0.86 nm the C_1_-OH releases N260 and binds to water (S14C Fig). Above 1 nm, D-ArbHO is too far from N260 and only interacts with water. All these events occur to overcome the energy barrier of the PMF (Fig 8B).

It has been shown that the pyranose ring of β-ArbOH is always linked to water molecules of the bulk solution, even at the binding site (S12A, S13A and S15A Figs). S15B–S15F Fig shows the conformations of D-ArbOH in the active site of metatyrosinase at the z-distances corresponding to the asterisks depicted in S14C Fig The participation of N260 in hydrogen bond interactions with D-ArbOH described above for S14 Fig can be seen in S15 Fig as a sequence of events. Note that the transition from the conformation of S15E Fig to that of S15F Fig would mean that the phenyl ring is rotated with respect to the oxane ring. At the same time, C_2_-OH…H_2_O…N260 is broken and C_1_-OH interacts directly with N260 through a hydrogen bond (S15 Fig) before D-ArbOH is drawn into to the bulk solution. These events correspond to the transition from the top of the energy barrier towards the water phase.

## Discussion

D-Arb is an apparent inhibitor of tyrosinase, which is why it is used as depigmenting agent in cosmetics and medicines. Its effectiveness as inhibitor of the activity of the enzyme on L-tyrosine and L-dopa [70] is higher than that of its precursors α and β-Arb, which were studied by our group in a previous work [26]. The aim of synthesising D-Arb was to obtain a more effective and less cytotoxic inhibitor than HQ and arbutins [28], although the SCCS decided in 2016 that D-Arb could only be used at up to 3% [31]. The kinetic study carried out by us supports the view that this compound is indeed effective at lower concentrations.

When the type of inhibition of tyrosinase by D-Arb was studied, an apparent competitive inhibition in both activities was observed (Fig 2A and 2B Inset). A mechanism to explain the action of tyrosinase on D-Arb in the presence of L-tyrosine is proposed in S16 Fig, while a possible mechanism for the action of tyrosinase on D-Arb and L-dopa is shown in S17 Fig Simulation of the mechanisms provided similar results (S18A and S18B Fig) to those obtained in Fig 2A and 2B, which are described in Table 1. The inhibition was seen to be competitive and the constant were $K_{I}^{\mathrm{app}}$ = 0.070 ± 0.004 and $K_{I}^{\mathrm{app}}$ = 0.062 ± 0.004 for the monophenolase and diphenolase activities, respectively.

In addition, the total oxygen consumption test (Fig 3A) demonstrated that D-Arb is a substrate of tyrosinase, since the absorbance and the reaction time of increase with increasing concentrations of this compound. Furthermore, a new maximum was obtained in the spectrophotometric recording when the enzyme acts on a mixture of TBC and D-Arb (Fig 3B).

D-Arb is a derived from a monophenol, so the presence of *E*_ox_ is necessary to run the catalytic cycle. This form of the enzyme can be generated by H_2_O_2_, AH_2_ and TBC (*o*-diphenol) and its catalytic activity is shown in Figs S2, S3, 3A and 3B respectively. Hence, various mechanisms are proposed to explain the action of tyrosinase in the presence of H_2_O_2_, AH_2_ and TBC in S19, S20 and S21 Figs, respectively. However, the most interesting observation is that tyrosinase can act on D-Arb without the addition of these compounds (Fig 4A).

Therefore, the mechanism proposed in Fig 5 shows that tyrosinase can act directly on D-Arb through its *E*_ox_ form, hydroxylating D-Arb to its *o*-diphenol and, subsequently, it does not release the *o*-diphenol, but transforms it to its quinone, so, the catalytic cycle is completed.

Note that this case differs from the action of tyrosinase on β-Arb [26]. In this previous work with β-Arb, we did not add *o*-diphenol, H_2_O_2_ or AH_2_ to the medium, but 3-methyl-2-benzothiazolinone hydrazone hydrochloride hydrate (MBTH). This potent nucleophile attacks the *o*-quinone and, after various reactions, leads to the accumulation of *o*-diphenol in the medium, which, in turn, activates the system, transforming *E*_m_ to *E*_d_, which becomes *E*_ox_ in the presence of O_2_ [71,72].

In the mechanism described above (Fig 5):

- There cannot be lag period in the accumulation of the product (P), such as that shown in Fig 4A.
- Activity should be increased in the presence of micromolar quantities of H_2_O_2_, since *E*_m_ becomes *E*_ox_, as demonstrated in Fig 6A. Note that the system already contains around 20% *E*_ox_ at the beggining, according to the results. This percentage agrees with the values (3–30%) described in the bibliography [73].
- The system responds to the addition of catalase. This enzyme breaks down H_2_O_2_, and so the *E*_ox_ becomes *E*_m_ and the activity disappears as is shown in Fig 6B.
- $V_{0}^{P}$ seems to have a linear dependence on the enzyme concentration and a hyperbolic dependence on the substrate concentration (Fig 4A).

The different tests mentioned above (“a-d”) can be carried out by numerical integration (S22, S23, S6 and S24 Figs, respectively) of the mechanism described in Fig 5. Thus, the obtained data confirm us the proposed mechanism, and by fitting the initial rate values by non-linear regression to Eq 7, we obtain the $k_{\mathrm{cat}}^{D-\mathrm{Arb}}$ and $K_{M}^{D-\mathrm{Arb}}$ values shown in Table 2. The $k_{\mathrm{cat}}^{D-\mathrm{Arb}}$ agrees with the chemical shift values of the carbon with the phenolic hydroxyl group and is very similar to those of α and β-Arb (Table 2). The $K_{M}^{D-\mathrm{Arb}}$ value is very low compared to that of β-Arb (Table 2), which can be explained by the hydrophobicity of the pyranose ring, which, in turn, increases the affinity of tyrosinase towards the substrate.

The characteristics shown by D-Arb acting as substrate of tyrosinase support our proposed mechanism. Regarding the monophenolase activity, we propose that the monophenol makes a nucleophilic attack on *E*_ox_ through the oxygen of OH group, transferring a proton to the peroxide group and giving rise to a hydroperoxide group, which, in turn, electrophilically attacks the *ortho* position of the benzene ring. The originated *o*-diphenol is in an axial/equatorial position and cannot be oxidized by the enzyme, since the oxygen orbitals are not coplanar with the copper atoms [42,51]. Subsequently, one of the copper bonds is broken and, in this way, when the *o*-diphenol is only bound to a copper atom, it can be released and the complex *E*_m_D-ArbOH can evolve to *E*_m_ + D-ArbOH or bind in diaxial position to be oxidized, giving rise to *E*_d_ + P. We emphasise the importance of this stage for the enzyme to act on monophenols.

D-Arb as a substrate differs from those described to date [18,26] since it involves the step from *E*_m_D-ArbOH to *E*_d_ + P as the only way the product can be generated. The above experiments and the previous works confirm the proposed mechanism (Fig 5) [74].

Therefore, in summary: a) *E*_ox_ is the only form able to act on D-Arb; b) H_2_O_2_ (μM) increases the concentration of *E*_ox_ and hence, the activity of the enzyme; c) pre-incubation with catalase decreases the activity of the enzyme. When D-Arb is compared with other alternative substrates of tyrosinase such as HQ or β-Arb, it can be observed that this compound does not need any compound (H_2_O_2_, AH_2_ or *o*-diphenol) to show activity.

The computational simulations showed that the N260 residue actively participates in the ligand binding process. Many authors have proposed N260 as a key residue in substrate binding in mushroom tyrosinase [75–82]. Besides, it has been demonstrated that an asparagine is essential to properly orientate the conserved water for proton transfer from a monophenol [69]. The presence of structural water in the copper centre of tyrosinase has been revealed in different crystallized tyrosinases and catechol oxidases, for example in sweet potato COx [83], *Streptomyces* tyrosinase [84], *Agaricus bisporus* tyrosinase [52], *Manduca sexta* tyrosinase [85] and tyrosinase from *Bacillus megaterium* [67].

The present study has demonstrated the importance of water molecules not only for their direct participation in substrate binding through aminoacid residues (i.e. N260), but also for acting as solvents facilitating or hindering substrate binding and/or dissociation. The simulations results point to the different behaviour of D-Arb and β-Arb in the active centre of tyrosinase as a result of their differences in polarity. Consequently, their interactions with the solvent might be a critical issue for defining the binding properties. It has been shown that D-Arb faces a barrier to scape of the binding pocket, partly composed of the solvent, which is mostly excluded from the binding cavity due to the presence of a hydrophobic environment created by D-Arb. This barrier is absent in the case of β-Arb, which is always in close contact with water molecules through hydrogen bond interactions. Taking into account all of the above it is feasible to propose that the high hydrophobicity of the oxane ring of D-Arb might contribute to keeping the *o*-diphenol product in the copper centre long enough to be further oxidized to the corresponding quinone. On the other hand, the o-diphenol product of β-Arb, with a polar pyranose ring, might be readily released from the active site to the water phase, avoiding oxidation to quinone.

In conclusion, this work demonstrates that D-Arb only apparently inhibits tyrosinase, since it is really an alternative substrate of the enzyme. This compound is the only substrate described to date that is hydroxylated by the enzyme without the generated *o*-diphenol being released to the medium. On the contrary, it is oxidized by the enzyme, which becomes *E*_d_ and releases the quinone (P). This imply that D-Arb should be used with caution as depigmenting agent, since the generated quinone could react with potent nucleophiles, such as cysteine and glutathione, disturbing the redox state of the cell.

## Supporting information

S1 Fig **13C NMR spectra of β-Arb (a) and D-Arb (b).** β-Arb (75 MHz, DMSO, 298K): δ 153.04 (C-O-C), **151.22 (C**_**Ar**_**-O-H)**, 118.53, 116.33, 102.59, 77.80, 77.48, 74.15, 70.66, 61.65. D-Arb (75 MHz, DMSO, 298K): δ 152.88 (C-O-C), **150.12 (C**_**Ar**_**-O-H)**, 118.73, 116.37, 97.72, 62.30, 30.93, 25.63, 19.64.
(TIF)

S2 Fig Action of tyrosinase on D-Arb in the presence of hydrogen peroxide. Spectrophotometric recordings of the action of tyrosinase on D-Arb in the presence of H_2_O_2_. The experimental conditions were [*E*]_0_ = 100 nM, [D-Arb]_0_ = 0.5 mM and [H_2_O_2_]_0_ = 20 mM. The spectrophotometric recordings were made every 60 seconds. **Inset.** Spectrophotometric recordings of the activity of different concentrations of tyrosinase on D-Arb in the presence of H_2_O_2_. The experimental conditions were [*E*]_0_ (nM) = (a) 25, (b) 50, (c) 75, (d) 100, (e) 150 and (f) 200; [D-Arb]_0_ = 0.5 mM and [H_2_O_2_]_0_ = 20 mM.
(TIF)

S3 Fig Action of tyrosinase on D-Arb in the presence of ascorbic acid. The experimental conditions were [*E*]_0_ = 50 nM, [D-Arb]_0_ = 0.1 mM and [AH_2_]_0_ = 0.2 mM. The spectrophotometric recordings were made every 2 minutes.
(TIF)

S4 Fig Tests to detect possible contaminations in D-Arb. Scans of (a) D-Arb (0.2 mM) and (b) an aliquot taken after D-Arb was filtered through a Sephadex G-25 column containing aluminium oxide (column preparation is described in Materials and Methods) eluted with ammonium acetate buffer at pH = 6.1. **Inset A.** Spectrophotometric recordings ((a) before and (b) after D-Arb passed through the column) at 485 nm of the oxidation of D-Arb by sodium periodate in excess to show that there is no contamination by *o*-diphenol. The experimental conditions were [D-Arb]_0_ = 0.2 mM and [NaIO_4_]_0_ = 0.5 mM. **Inset B.** Activity of tyrosinase on the eluted D-Arb. The experimental conditions were [D-Arb]_0_ = 0.25 mM and [*E*]_0_ = 50 nM.
(TIF)

S5 Fig Determination of the molar absorptivity coefficient of quinone of D-Arb. Final absorbance at 485 nm of the spectrophotometric recordings of the action of tyrosinase on different concentrations of D-Arb until its consumption. The experimental conditions were: [H_2_O_2_]_0_ = 5 mM and [*E*]_0_ = 200 nM. **Inset.** Spectrophotometric recordings of the main figure. [D-Arb]_0_ (mM): (a) 0.05, (b) 0.10, (c) 0.15 and (d) 0.20.
(TIF)

S6 Fig Simulation of the action of tyrosinase on D-Arb in the presence of catalase. Representation of initial rate values of tyrosinase on D-Arb calculated from the simulated progress curves obtained through numerical integration of the set of differential equations corresponding to the mechanism shown in Fig 5, adding the action of catalase. The simulated conditions were [*E*]_0_ = 500 nM, [*E*_ox_]_0_ = 0.2 x [*E*]_0_, [*E*_m_]_0_ = 0.8 x [*E*]_0_; [D-Arb]_0_ = 0.2 mM and [O_2_]_0_ = 0.26 mM. The rate constants were: *k*_8_ = 2.3 x 10^8^ M^-1^ s^-1^, *k*_-8_ = 1.07 x 10^3^ s^-1^, *k*_9_ = 1.6 x 10^5^ M^-1^ s^-1^, *k*_-9_ = 3.8 s^-1^, *k*_10_ = 1.5 s^-1^, *k*_11_ = 400 s^-1^, *k*_12_ = 1.6 x 10^5^ M^-1^ s^-1^, *k*_-12_ = 3.8 s^-1^, *k*_15_ = 2 x 10^6^ M^-1^ s^-1^, *k*_-15_ = 10 s^-1^, *k*_16_ = 6.6 x 10^−4^ s^-1^.
(TIF)

S7 Fig Schematic representation of the action mechanism of tyrosinase on D-Arb adding the possibility of D-ArbOH being released to the medium and oxidized immediately. (TIF)

S8 Fig Simulation of the action of tyrosinase on D-Arb adding the possibility of D-ArbOH being released to the medium and oxidized immediately. Representation of the accumulation of P due to the action of tyrosinase on D-Arb calculated from the simulated progress curves obtained through numerical integration of the set of differential equations corresponding to the mechanism shown in Fig 5, adding the possibility of D-ArbOH being released to the medium and oxidized immediately with the rate constants *k*_*-*13_ and *k*_19_, respectively (S7 Fig). The simulated conditions were [*E*]_0_ = 750 nM, [*E*_ox_]_0_ = 0.2 x [*E*]_0_, [*E*_m_]_0_ = 0.8 x [*E*]_0_; [H_2_O_2_]_0_ = 1.25 μM, [D-Arb]_0_ = 0.4 mM and [O_2_]_0_ = 0.26 mM. The rate constants were: *k*_8_ = 2.3 x 10^8^ M^-1^ s^-1^, *k*_-8_ = 1.07 x 10^3^ s^-1^, *k*_9_ = 1.6 x 10^5^ M^-1^ s^-1^, *k*_-9_ = 3.8 s^-1^, *k*_10_ = 1.5 s^-1^, *k*_11_ = 400 s^-1^, *k*_12_ = 1.6 x 10^5^ M^-1^ s^-1^, *k*_-12_ = 3.8 s^-1^, *k*_13_ = 10^5^ M^-1^ s^-1^, *k*_*-*13_ = 3 s^-1^, *k*_15_ = 2 x 10^6^ M^-1^ s^-1^, *k*_*-*15_ = 10 s^-1^, *k*_19_ = 3 x 10^4^ s^-1^. **Inset.** Concentrations of *E*_ox_ and *E*_m_ with time in the simulated assay described in the main figure.
(TIF)

S9 Fig Schematic representation of the action mechanism of tyrosinase on D-Arb adding the possibility of D-ArbOH being released to the medium and bond to *E*_m_ and *E*_ox_. (TIF)

S10 Fig Simulation of the action of tyrosinase on D-Arb adding the possibility of D-ArbOH being released to the medium and bond to *E*_m_ and *E*_ox_. Representation of the accumulation of P due to the action of tyrosinase on D-Arb calculated from the simulated progress curves obtained through numerical integration of the set of differential equations corresponding to the mechanism shown in Fig 5, adding the possibility of D-ArbOH being released to the medium from the complex *E*_m_D-ArbOH with the rate constant *k*_*-*13_ and bond to *E*_m_ and *E*_ox_ with the rate constants *k*_13_ and *k*_17_, respectively (S9 Fig). The simulated conditions were [*E*]_0_ = 750 nM, [*E*_ox_]_0_ = 0.2 x [*E*]_0_, [*E*_m_]_0_ = 0.8 x [*E*]_0_; [H_2_O_2_]_0_ = 1.25 μM, [D-Arb]_0_ = 0.4 mM and [O_2_]_0_ = 0.26 mM. The rate constants were: *k*_8_ = 2.3 x 10^8^ M^-1^ s^-1^, *k*_-8_ = 1.07 x 10^3^ s^-1^, *k*_9_ = 1.6 x 10^5^ M^-1^ s^-1^, *k*_-9_ = 3.8 s^-1^, *k*_10_ = 1.5 s^-1^, *k*_11_ = 400 s^-1^, *k*_12_ = 1.6 x 10^5^ M^-1^ s^-1^, *k*_-12_ = 3.8 s^-1^, *k*_13_ = 10^5^ M^-1^ s^-1^, *k*_*-*13_ = 3 s^-1^, *k*_15_ = 2 x 10^6^ M^-1^ s^-1^, *k*_-15_ = 10 s^-1^, *k*_17_ = 10^5^ M^-1^ s^-1^,*k*_*-*17_ = 3 s^-1^, *k*_18_ = 400 s^-1^.
(TIF)

S11 Fig Simulation of the action of tyrosinase on D-Arb according to Fig 5. Representation of the accumulation of P due to the action of tyrosinase on D-Arb calculated from the simulated progress curves obtained through numerical integration of the set of differential equations corresponding to the mechanism shown in Fig 5. The simulated conditions were [*E*]_0_ = 750 nM, [*E*_ox_]_0_ = 0.2 x [*E*]_0_, [*E*_m_]_0_ = 0.8 x [*E*]_0_; [H_2_O_2_]_0_ = 1.25 μM, [D-Arb]_0_ = 0.4 mM and [O_2_]_0_ = 0.26 mM. The rate constants were: *k*_8_ = 2.3 x 10^8^ M^-1^ s^-1^, *k*_-8_ = 1.07 x 10^3^ s^-1^, *k*_9_ = 1.6 x 10^5^ M^-1^ s^-1^, *k*_-9_ = 3.8 s^-1^, *k*_10_ = 1.5 s^-1^, *k*_11_ = 400 s^-1^, *k*_12_ = 1.6 x 10^5^ M^-1^ s^-1^, *k*_-12_ = 3.8 s^-1^, *k*_13_ = 10^5^ M^-1^ s^-1^, *k*_*-*13_ = 3 s^-1^, *k*_15_ = 2 x 10^6^ M^-1^ s^-1^, *k*_-15_ = 10 s^-1^.
(TIF)

S12 Fig **Surface representation of the configuration poses of β-Arb (A) and D-Arb (B) corresponding to Fig 7.** View from the water phase to the atoms copper buried in the protein structure.
(TIF)

S13 Fig Water molecules distribution in the cavity of the ligand binding pocket along z-axis oriented from the copper atoms to the water phase. The ligand configurations are scaled and placed at the binding pose for. Copper atoms are located at about 4.2 nm z-distance. (A) β-ArbOH, (B) D-ArbOH.
(TIF)

S14 Fig Hydrogen bonds of *o*-diphenols corresponding to the conformations distribution of the PMF curves (Fig 8B). (A) Hydrogen bonds of β-ArbOH (upper trace) and D-ArbOH (lower trace) with water. (B) Hydrogen bonds of *o*-phenol group (C_2_-OH) (solid line) and C_1_-OH (dashed line) hydroxyl groups of D-ArbOH with water. (C) Hydrogen bonds of C_2_-OH (solid line) and C_1_-OH (dashed line) hydroxyl groups of D-ArbOH with N260 residue. The asterisks mark the positions of the conformation poses shown in S15B–S15F Fig.
(TIF)

S15 Fig Conformational poses of *o*-diphenols at different positions of z-axis. (A) β-ArbOH at the copper centre binding site. From (B) to (F) conformational structures of D-ArbOH at the positions marked as asterisks in S14C Fig. The atom colors are as follows: red = oxygen, blue = nitrogen, brown (spheres) = copper, green = carbon and white = hydrogen. In yellow dashed lines possible hydrogen bonds interactions are shown. Only the most relevant residues are depicted.
(TIF)

S16 Fig Schematic representation of the kinetic mechanism of the action of tyrosinase on L-tyrosine in the presence of D-Arb. M = monophenol (L-tyrosine), D = *o*-diphenol (L-dopa), Q = *o*-dopaquinone, Cr = dopachrome.
(TIF)

S17 Fig Schematic representation of the kinetic mechanism of the action of tyrosinase on L-dopa in the presence of D-Arb. (TIF)

S18 Fig Simulation of the inhibition of the monophenolase activity of tyrosinase by D-Arb. **A.** Representation of initial rate values of tyrosinase on L-tyrosine in the absence (●) and presence (▲) of D-Arb calculated from the simulated progress curves obtained through numerical integration of the set of differential equations corresponding to the mechanism shown in S16 Fig. The simulated conditions were [*E*]_0_ = 700 nM, [*E*_ox_]_0_ = 0.2 x [*E*]_0_, [*E*_m_]_0_ = 0.8 x [*E*]_0_; [D-Arb]_0_ = 0.2 mM, [O_2_]_0_ = 0.26 mM and R = [L-dopa]_0_ / [L-tyrosine]_0_ = 0.042. The rate constants were: *k*_1_ = 2 x 10^5^ M^-1^ s^-1^, *k*_-1_ = 10 s^-1^, *k*_2_ = 5 x 10^5^ M^-1^ s^-1^, *k*_-2_ = 10 s^-1^, *k*_3_ = 900 s^-1^, *k*_4_ = 4.8 x 10^4^ M^-1^ s^-1^, *k*_-4_ = 0.5 s^-1^, *k*_5_ = 12 s^-1^, *k*_6_ = 2.16 x 10^5^ M^-1^ s^-1^, *k*_-6_ = 10 s^-1^, *k*_7_ = 108 s^-1^, *k*_8_ = 2.3 x 10^8^ M^-1^ s^-1^, *k*_-8_ = 1.07 x 10^3^ s^-1^, *k*_9_ = 1.6 x 10^5^ M^-1^ s^-1^, *k*_-9_ = 3.8 s^-1^, *k*_10_ = 1.5 s^-1^, *k*_11_ = 400 s^-1^, *k*_12_ = 1.6 x 10^5^ M^-1^ s^-1^, *k*_-12_ = 3.8 s^-1^, *k*_14_ = 10 s^-1^. **Inset.** Graphical representation of the Lineweaver–Burk equation showing the simulated inhibition of the monophenolase activity of tyrosinase in the absence (●) and presence (▲) of D-Arb. The experimental conditions were the same as those of the main figure. **B. Simulation of the inhibition of the diphenolase activity of tyrosinase by D-Arb.** Representation of initial rate values of tyrosinase on L-dopa in the absence (●) and presence (▲) of D-Arb calculated from the simulated progress curves obtained through numerical integration of the set of differential equations corresponding to the mechanism shown in S17 Fig. The simulated conditions were [*E*]_0_ = 700 nM, [*E*_ox_]_0_ = 0.2 x [*E*]_0_, [*E*_m_]_0_ = 0.8 x [*E*]_0_; [D-Arb]_0_ = 0.2 mM and [O_2_]_0_ = 0.26 mM. The rate constants were: *k*_2_ = 5 x 10^5^ M^-1^ s^-1^, *k*_-2_ = 10 s^-1^, *k*_3_ = 900 s^-1^, *k*_4_ = 4.8 x 10^4^ M^-1^ s^-1^, *k*_-4_ = 0.5 s^-1^, *k*_5_ = 12 s^-1^, *k*_6_ = 2.16 x 10^5^ M^-1^ s^-1^, *k*_-6_ = 10 s^-1^, *k*_7_ = 108 s^-1^, *k*_8_ = 2.3 x 10^8^ M^-1^ s^-1^, *k*_-8_ = 1.07 x 10^3^ s^-1^, *k*_9_ = 1.6 x 10^5^ M^-1^ s^-1^, *k*_-9_ = 3.8 s^-1^, *k*_10_ = 1.5 s^-1^, *k*_11_ = 400 s^-1^, *k*_12_ = 1.6 x 10^5^ M^-1^ s^-1^, *k*_-12_ = 3.8 s^-1^, *k*_14_ = 10 s^-1^. **Inset.** Graphical representation of the Lineweaver–Burk equation showing the simulated inhibition of the diphenolase activity of tyrosinase in the absence (●) and presence (▲) of D-Arb. The experimental conditions were the same as in the main figure.
(TIF)

S19 Fig Schematic representation of the kinetic mechanism of the action of tyrosinase on D-Arb in the presence of H_2_O_2_. (TIF)

S20 Fig Schematic representation of the kinetic mechanism of the action of tyrosinase on D-Arb in the presence of AH_2_. (TIF)

S21 Fig Schematic representation of the kinetic mechanism of the action of tyrosinase on D-Arb in the presence of TBC. (TIF)

S22 Fig Simulated recordings of the action of tyrosinase on D-Arb. Accumulation of P due to the action (with no lag period) of tyrosinase on different concentrations of D-Arb calculated from the simulated progress curves obtained through numerical integration of the set of differential equations corresponding to the mechanism shown in Fig 5. The simulated conditions were [*E*]_0_ = 700 nM, [*E*_ox_]_0_ = 0.2 x [*E*]_0_, [*E*_m_]_0_ = 0.8 x [*E*]_0_; [D-Arb]_0_ (mM) = (a) 0.05, (b) 0.10, (c) 0.25, (d) 0.40, (e) 0.70 and (f) 1; [H_2_O_2_]_0_ = 1.25 μM and [O_2_]_0_ = 0.26 mM. The rate constants were: *k*_8_ = 2.3 x 10^8^ M^-1^ s^-1^, *k*_-8_ = 1.07 x 10^3^ s^-1^, *k*_9_ = 1.6 x 10^5^ M^-1^ s^-1^, *k*_-9_ = 3.8 s^-1^, *k*_10_ = 1.5 s^-1^, *k*_11_ = 400 s^-1^, *k*_12_ = 1.6 x 10^5^ M^-1^ s^-1^, *k*_-12_ = 3.8 s^-1^, *k*_15_ = 2 x 10^6^ M^-1^ s^-1^, *k*_-15_ = 10 s^-1^.
(TIF)

S23 Fig Simulation of the action of tyrosinase on D-Arb after a pre-incubation of the enzyme with hydrogen peroxide. Representation of initial rate values of tyrosinase on D-Arb after pre-incubation with different concentrations of H_2_O_2_ (μM) during a minute, calculated from the simulated progress curves obtained through numerical integration of the set of differential equations corresponding to the mechanism shown in Fig 5. The simulated conditions were [*E*]_0_ = 500 nM, [*E*_ox_]_0_ = 0.2 x [*E*]_0_, [*E*_m_]_0_ = 0.8 x [*E*]_0_; [H_2_O_2_]_0_ = 1.25 μM and [O_2_]_0_ = 0.26 mM. After the pre-incubation, the final values of [*E*_m_], [*E*_ox_] and [H_2_O_2_] were used to simulate the activity of the enzyme on D-Arb (0.2 mM). The rate constants were: *k*_8_ = 2.3 x 10^8^ M^-1^ s^-1^, *k*_-8_ = 1.07 x 10^3^ s^-1^, *k*_9_ = 1.6 x 10^5^ M^-1^ s^-1^, *k*_-9_ = 3.8 s^-1^, *k*_10_ = 1.5 s^-1^, *k*_11_ = 400 s^-1^, *k*_12_ = 1.6 x 10^5^ M^-1^ s^-1^, *k*_-12_ = 3.8 s^-1^, *k*_15_ = 2 x 10^6^ M^-1^ s^-1^, *k*_-15_ = 10 s^-1^.
(TIF)

S24 Fig Simulated action of tyrosinase on D-Arb with different concentrations of enzyme and substrate. Representation of initial rate values of tyrosinase on D-Arb with different concentrations of enzyme calculated from the simulated progress curves obtained through numerical integration of the set of differential equations corresponding to the mechanism shown in Fig 5. The simulated conditions were [*E*_ox_]_0_ = 0.2 x [*E*]_0_, [*E*_m_]_0_ = 0.8 x [*E*]_0_, being [*E*]_0_ (nM) = 0, 50,100, 250, 500 and 750, respectively; [H_2_O_2_]_0_ = 1.25 μM, [D-Arb]_0_ = 0.2 mM and [O_2_]_0_ = 0.26 mM. The rate constants were: *k*_8_ = 2.3 x 10^8^ M^-1^ s^-1^, *k*_-8_ = 1.07 x 10^3^ s^-1^, *k*_9_ = 1.6 x 10^5^ M^-1^ s^-1^, *k*_-9_ = 3.8 s^-1^, *k*_10_ = 1.5 s^-1^, *k*_11_ = 400 s^-1^, *k*_12_ = 1.6 x 10^5^ M^-1^ s^-1^, *k*_-12_ = 3.8 s^-1^, *k*_15_ = 2 x 10^6^ M^-1^ s^-1^, *k*_-15_ = 10 s^-1^. **Inset.** Representation of initial rate values of tyrosinase on different concentrations of D-Arb calculated from the simulated progress curves obtained through numerical integration of the set of differential equations corresponding to the mechanism shown in Fig 5. The simulated conditions were the same as those of the main figure.
(TIF)

S1 File Kinetic analysis. (DOCX)

S2 File Conditions of simulation assays. (DOCX)
