# Supplementary figures and images for: Structural and kinetic considerations on the catalysis of deoxyarbutin by tyrosinase
Source: PLoS One. 2017 Nov 14;12(11):e0187845. doi: 10.1371/journal.pone.0187845 (PMC5685642; doi:10.1371/journal.pone.0187845)

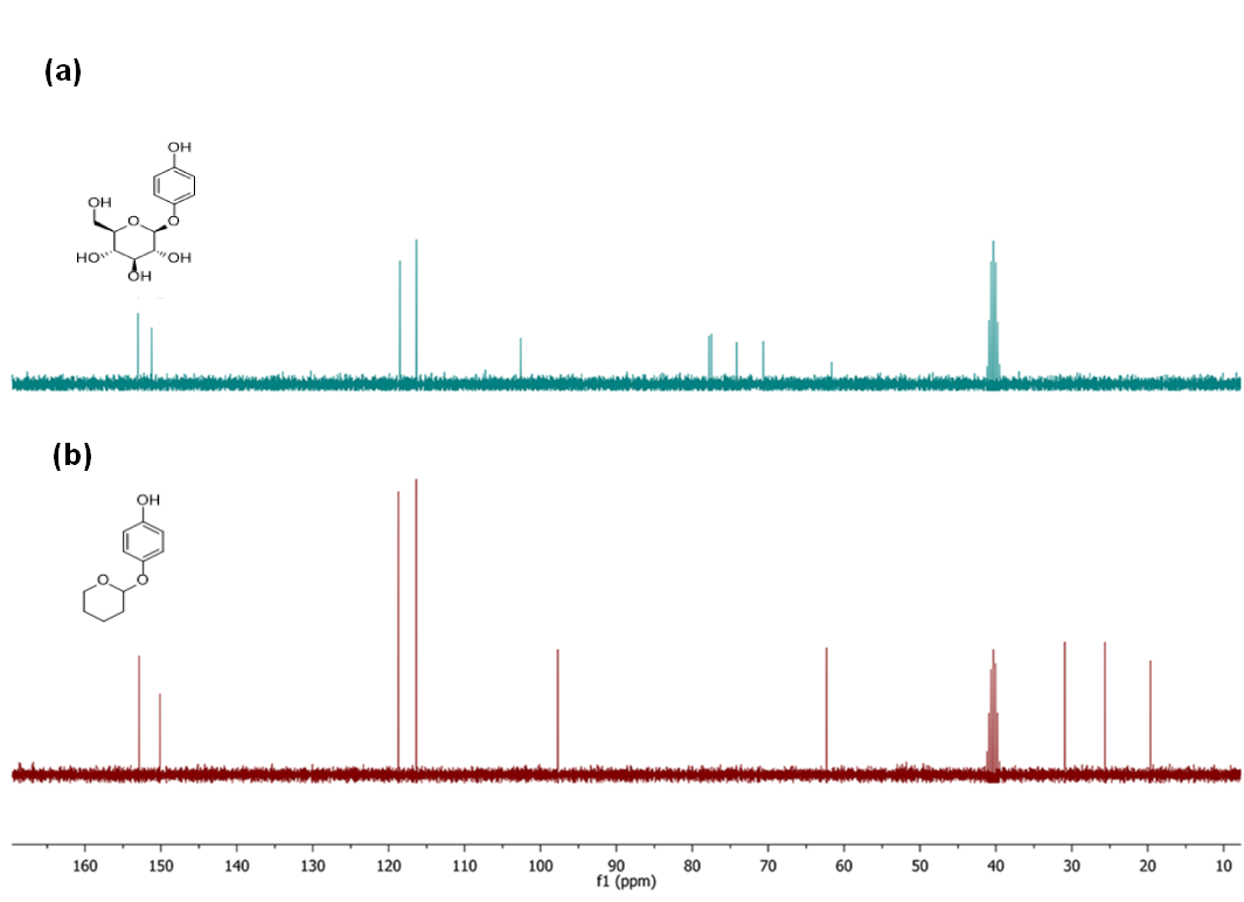

Supplement: S1 Fig — 13C NMR spectra of β-Arb (a) and D-Arb (b). β-Arb (75 MHz, DMSO, 298K): δ 153.04 (C-O-C), 151.22 (CAr-O-H), 118.53, 116.33, 102.59, 77.80, 77.48, 74.15, 70.66, 61.65. D-Arb (75 MHz, DMSO, 298K): δ 152.88 (C-O-C), 150.12 (CAr-O-H), 118.73, 116.37, 97.72, 62.30, 30.93, 25.63, 19.64. (TIF) [file pone.0187845.s001.tif]

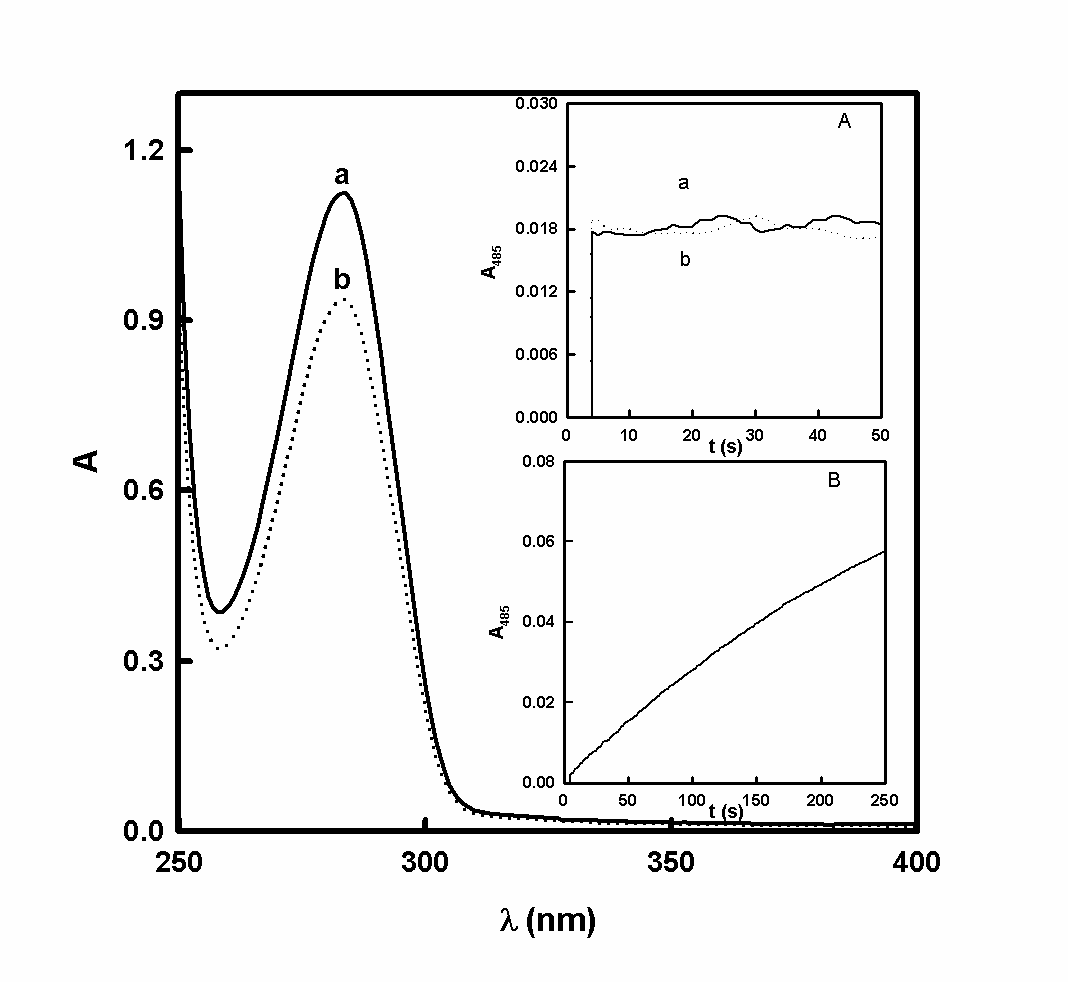

Supplement: S4 Fig — Scans of (a) D-Arb (0.2 mM) and (b) an aliquot taken after D-Arb was filtered through a Sephadex G-25 column containing aluminium oxide (column preparation is described in Materials and Methods) eluted with ammonium acetate buffer at pH = 6.1. Inset A. Spectrophotometric recordings ((a) before and (b) after D-Arb passed through the column) at 485 nm of the oxidation of D-Arb by sodium periodate in excess to show that there is no contamination by o-diphenol. The experimental conditions were [D-Arb]0 = 0.2 mM and [NaIO4]0 = 0.5 mM. Inset B. Activity of tyrosinase on the eluted D-Arb. The experimental conditions were [D-Arb]0 = 0.25 mM and [E]0 = 50 nM. (TIF) [file pone.0187845.s004.tif]

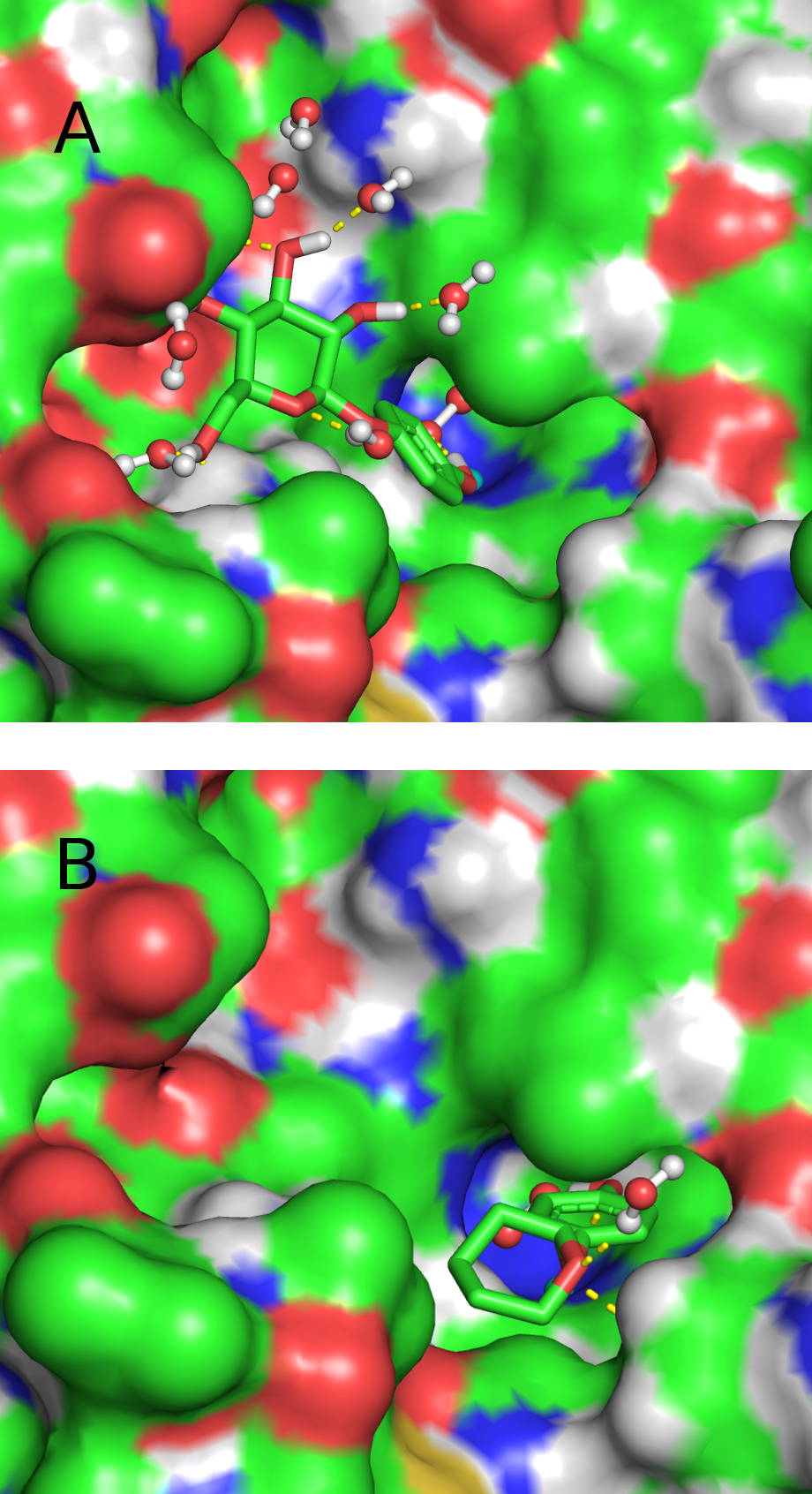

Supplement: S12 Fig — Surface representation of the configuration poses of β-Arb (A) and D-Arb (B) corresponding to Fig 7. View from the water phase to the atoms copper buried in the protein structure. (TIF) [file pone.0187845.s012.tif]

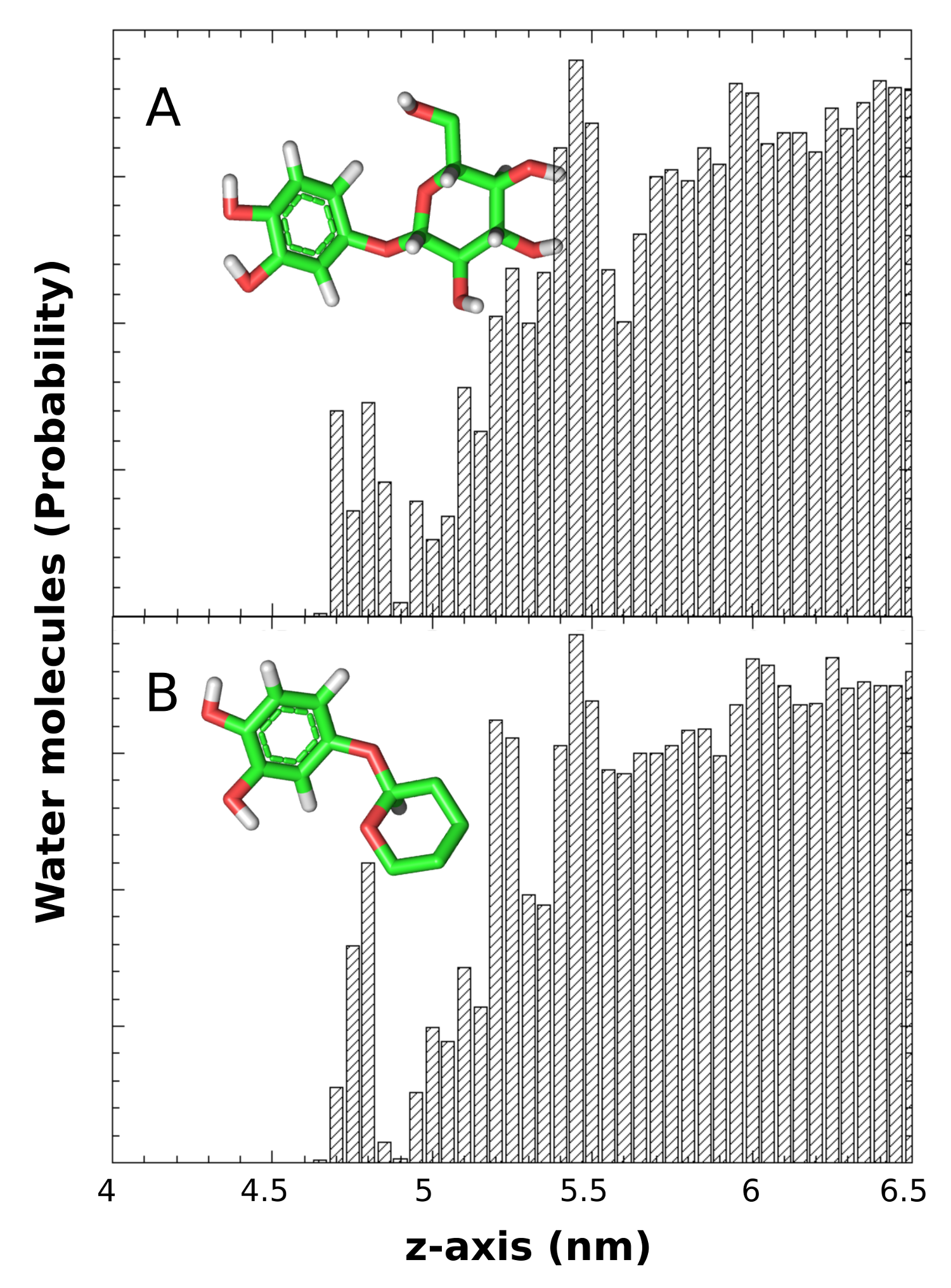

Supplement: S13 Fig — The ligand configurations are scaled and placed at the binding pose for. Copper atoms are located at about 4.2 nm z-distance. (A) β-ArbOH, (B) D-ArbOH. (TIF) [file pone.0187845.s013.tif]

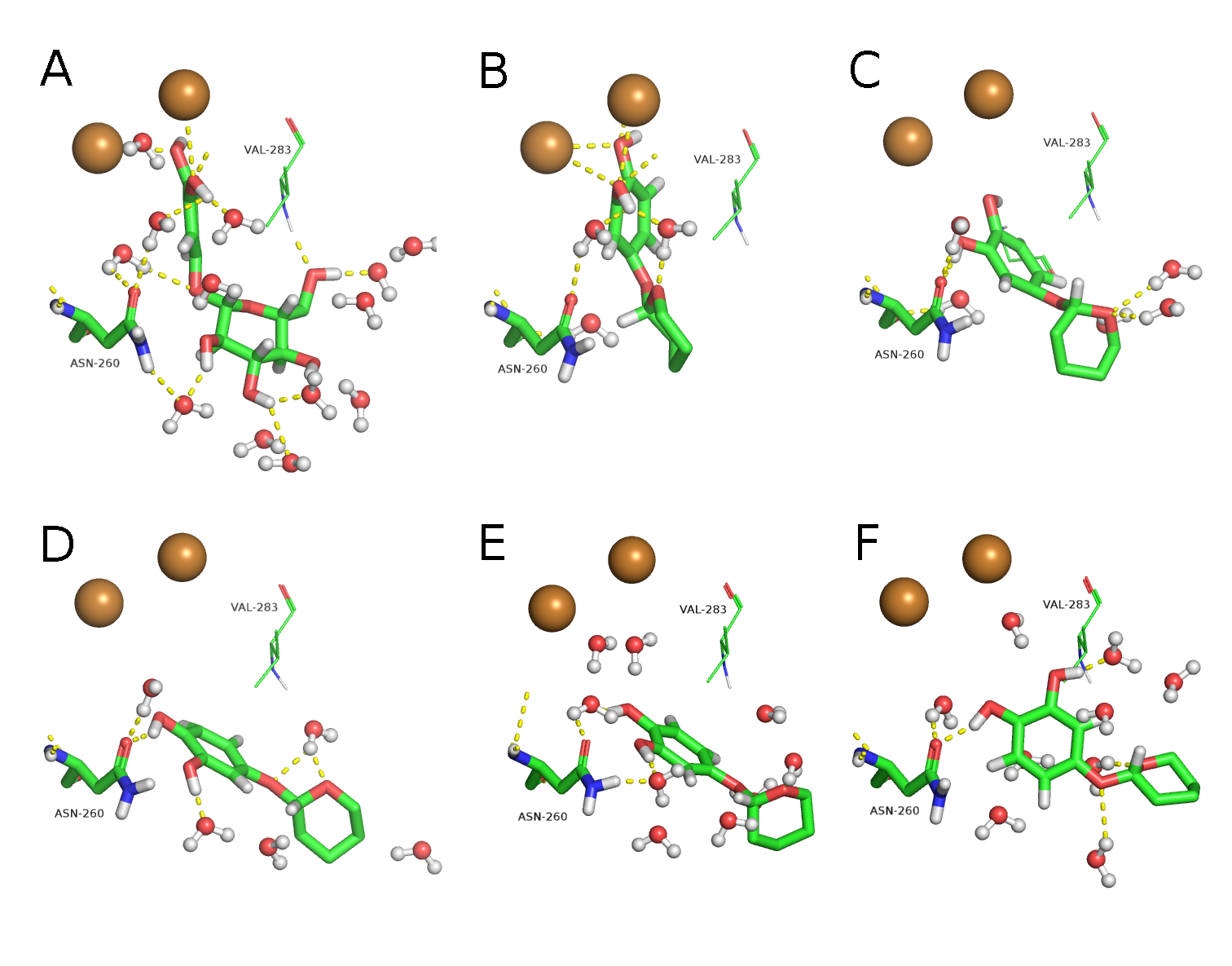

Supplement: S15 Fig — (A) β-ArbOH at the copper centre binding site. From (B) to (F) conformational structures of D-ArbOH at the positions marked as asterisks in S14C Fig. The atom colors are as follows: red = oxygen, blue = nitrogen, brown (spheres) = copper, green = carbon and white = hydrogen. In yellow dashed lines possible hydrogen bonds interactions are shown. Only the most relevant residues are depicted. (TIF) [file pone.0187845.s015.tif]

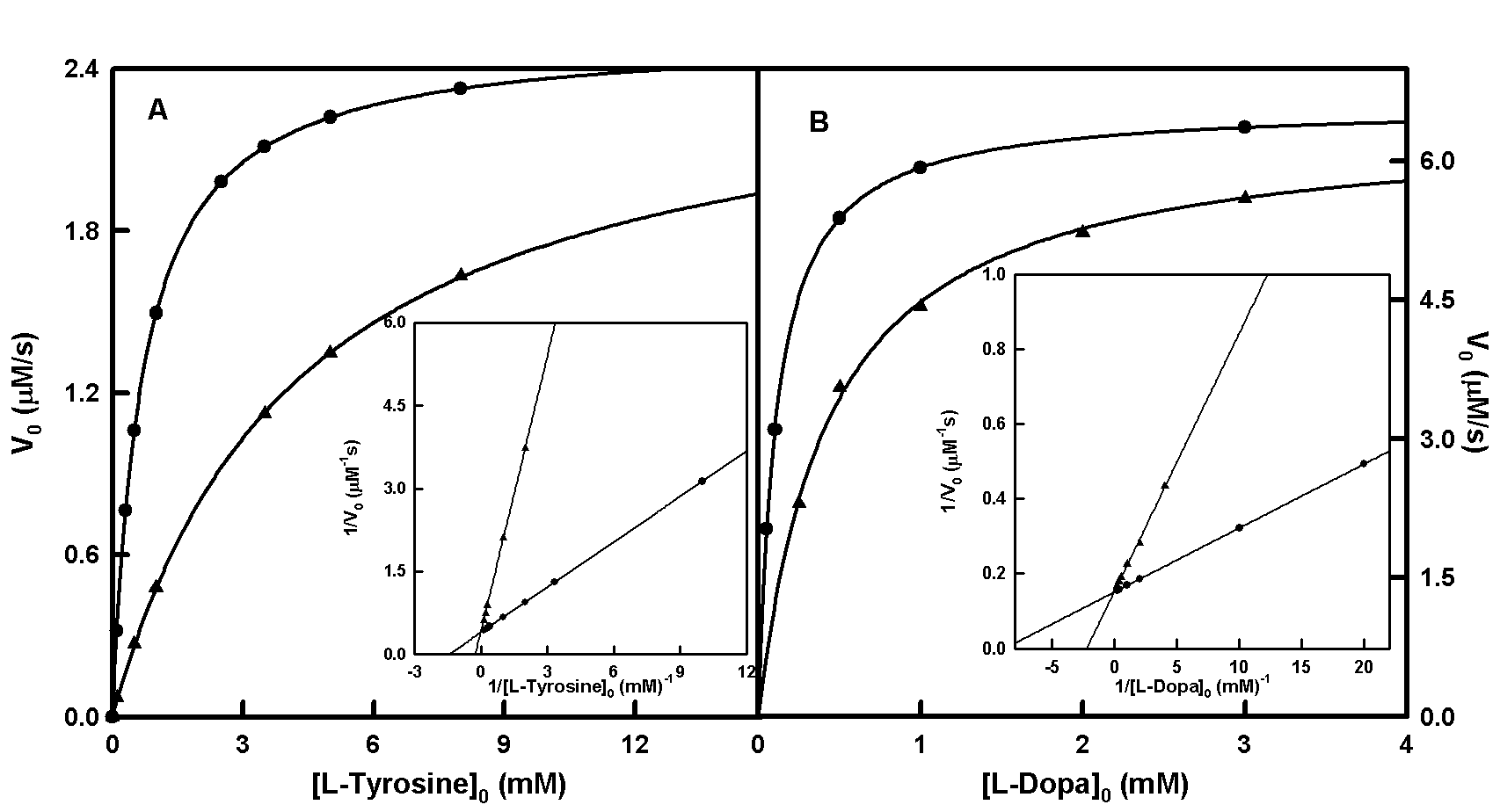

Supplement: S18 Fig — A. Representation of initial rate values of tyrosinase on L-tyrosine in the absence (●) and presence (▲) of D-Arb calculated from the simulated progress curves obtained through numerical integration of the set of differential equations corresponding to the mechanism shown in S16 Fig. The simulated conditions were [E]0 = 700 nM, [Eox]0 = 0.2 x [E]0, [Em]0 = 0.8 x [E]0; [D-Arb]0 = 0.2 mM, [O2]0 = 0.26 mM and R = [L-dopa]0 / [L-tyrosine]0 = 0.042. The rate constants were: k1 = 2 x 10^5^ M-1 s-1, k-1 = 10 s-1, k2 = 5 x 10^5^ M-1 s-1, k-2 = 10 s-1, k3 = 900 s-1, k4 = 4.8 x 10^4^ M-1 s-1, k-4 = 0.5 s-1, k5 = 12 s-1, k6 = 2.16 x 10^5^ M-1 s-1, k-6 = 10 s-1, k7 = 108 s-1, k8 = 2.3 x 10^8^ M-1 s-1, k-8 = 1.07 x 103 s-1, k9 = 1.6 x 10^5^ M-1 s-1, k-9 = 3.8 s-1, k10 = 1.5 s-1, k11 = 400 s-1, k12 = 1.6 x 10^5^ M-1 s-1, k-12 = 3.8 s-1, k14 = 10 s-1. Inset. Graphical representation of the Lineweaver–Burk equation showing the simulated inhibition of the monophenolase activity of tyrosinase in the absence (●) and presence (▲) of D-Arb. The experimental conditions were the same as those of the main figure. B. Simulation of the inhibition of the diphenolase activity of tyrosinase by D-Arb. Representation of initial rate values of tyrosinase on L-dopa in the absence (●) and presence (▲) of D-Arb calculated from the simulated progress curves obtained through numerical integration of the set of differential equations corresponding to the mechanism shown in S17 Fig. The simulated conditions were [E]0 = 700 nM, [Eox]0 = 0.2 x [E]0, [Em]0 = 0.8 x [E]0; [D-Arb]0 = 0.2 mM and [O2]0 = 0.26 mM. The rate constants were: k2 = 5 x 10^5^ M-1 s-1, k-2 = 10 s-1, k3 = 900 s-1, k4 = 4.8 x 10^4^ M-1 s-1, k-4 = 0.5 s-1, k5 = 12 s-1, k6 = 2.16 x 10^5^ M-1 s-1, k-6 = 10 s-1, k7 = 108 s-1, k8 = 2.3 x 10^8^ M-1 s-1, k-8 = 1.07 x 103 s-1, k9 = 1.6 x 10^5^ M-1 s-1, k-9 = 3.8 s-1, k10 = 1.5 s-1, k11 = 400 s-1, k12 = 1.6 x 10^5^ M-1 s-1, k-12 = 3.8 s-1, k14 = 10 s-1. Inset. Graphical representation of the Lineweaver–Burk equation showin [file pone.0187845.s018.TIF]
