# Supplementary material for: Structural and kinetic considerations on the catalysis of deoxyarbutin by tyrosinase
Source: PLoS One. 2017 Nov 14;12(11):e0187845. doi: 10.1371/journal.pone.0187845 (PMC5685642; doi:10.1371/journal.pone.0187845)
Supplement: S1 File — (DOCX) [file pone.0187845.s025.docx]

**Kinetic analysis**

The mechanisms described in S16 and S17 Figs reflect the action of tyrosinase on its physiological substrates L-tyrosine (M) and L-dopa (D), in the presence of D-Arb, which behaves as a substrate. The rate equations were obtained by means of the ALBASS program, designed specifically to obtain the rate equations for enzymatic reactions in steady-state [[1](#_ENREF_1)].

In the mechanisms described in S16 and S17 Figs, the following notation was used:

*E*_m_ metatyrosinase

*E*_d_ deoxytyrosinase

*E*_ox_ oxytyrosinase

*E*_m_D metatyrosinase/L-dopa complex

*E*_ox_D oxytyrosinase/L-dopa complex

*E*_m_D-ArbOH metatyrosinase/*o*-diphenol complex derived from D-Arb

*E*_ox_D-ArbOH oxytyrosinase/*o*-diphenol complex derived from D-Arb

*E*_m_M metatyrosinase/L-tyrosine complex

*E*_ox_M oxytyrosinase/L-tyrosine complex

[*E*]_0_ initial concentration of tyrosinase

[D] L-dopa

[D]_0_ initial concentration in steady-state

D-Arb deoxyarbutin

[D-Arb]_0_ initial concentration of deoxyarbutin

M L-tyrosine

[M]_0_ initial concentration of L-tyrosine

D-ArbOH *o*-diphenol corresponding to D-Arb

Q *o*-quinone corresponding to L-dopa

P quinone corresponding to D-ArbOH

[P] instantaneous concentration of P

Cr dopachrome

[Cr] instantaneous concentration of dopachrome

$V_{0}^{\text{Q}}$ initial rate of tyrosinase acting on L-dopa (D) or L-tyrosine (M) calculated by measuring Q

$V_{0}^{\text{Cr}}$ initial rate of tyrosinase acting on L-dopa (D) or L-tyrosine (M) calculated by measuring Cr ($V_{0}^{\text{Cr}}$=$V_{0}^{\text{Q}}$/2)

$V_{0}^{\text{P}}$ initial rate of tyrosinase acting on deoxyarbutin calculated by measuring P

$V_{0}^{\text{(Cr+P)(D)(D-Arb)}}$ initial rate of tyrosinase acting on L-dopa (D) and D-Arb calculated by measuring Cr and P

$V_{0}^{\text{(Cr+P)(M)(D)(D-Arb)}}$ initial rate of tyrosinase acting on L-tyrosine (M), L-dopa (D) and D-Arb calculated by measuring Cr and P

R ratio between [D]_ss_ and [M]_ss_, R = [D]_ss_/[M]_ss_ [D]_ss_/[M]_0_

*k_i_* rate constants in the catalytic route (*i*<*k*_14_)

Applying the steady-state approximation to the mechanism described in S16 Fig for the activity of tyrosinase on monophenols (M) and *o*-diphenols (D) in the presence of D-Arb provides the following expression for the products formation rate [[1](#_ENREF_1)]:

$\text{V}_{0}^{(Cr+P)(M)(D)(D\text{-}Arb)}=\frac{\left[\begin{matrix} \text{α}_{1}\left[O_{2}\right]_{0}\left[D\right]_{0}\left[M\right]_{0}+\text{α}_{2}\left[O_{2}\right]_{0}\left[D\right]_{0}^{2}+ \\ +2\text{α}_{3}\left[O_{2}\right]_{0}\left[D\text{-}Arb\right]_{0}\left[D\right]_{0} \end{matrix}\right]\left[\text{E}\right]_{0}}{\begin{matrix} 2(\text{β}_{1}\left[D\right]_{0} +\text{β}_{2}\left[D\right]_{0}\left[M\right]_{0}\text{+β}_{3}\left[D\right]_{0}^{2}\text{+β}_{4}\left[O_{2}\right]_{0}\left[M\right]_{0}\text{+} \\ \text{+β}_{5}\left[O_{2}\right]_{0}\left[D\right]_{0}\text{+β}_{6}\left[D\text{-}Arb\right]_{0}\left[D\right]_{0}\text{+β}_{7}\left[O_{2}\right]_{0}\left[M\right]_{0}^{2}\text{+} \\ \text{+β}_{8}\left[O_{2}\right]_{0}\left[D\right]_{0}\left[M\right]_{0}\text{+β}_{9}\left[O_{2}\right]_{0}\left[D\right]_{0}^{2}\text{+} \\ \text{+β}_{10}\left[D\text{-}Arb\right]_{0}\left[O_{2}\right]_{0}\left[M\right]_{0}\text{+β}_{11}\left[D\text{-}Arb\right]_{0}\left[O_{2}\right]_{0}\left[D\right]_{0}) \end{matrix}}$ (S1)

The oxygen concentration is saturating [[2-4](#_ENREF_2)], so [O_2_]_0_ 🡪 ∞ and the following expression is obtained:

$\text{V}_{0}^{(Cr+P)(M)(D)(D\text{-}Arb)}=\frac{\left[\text{α}_{1}\left[M\right]_{0}\left[D\right]_{0}+\text{α}_{2}\left[D\right]_{0}^{2}+2\text{α}_{3}\left[D\text{-}Arb\right]_{0}\left[D\right]_{0}\right]\left[\text{E}\right]_{0}}{\begin{matrix} 2(\text{β}_{4}\left[M\right]_{0} +\text{β}_{5}\left[D\right]_{0}\text{+β}_{7}\left[M\right]_{0}^{2}\text{+β}_{8}\left[M\right]_{0}\left[D\right]_{0}\text{+} \\ \text{+β}_{9}\left[D\right]_{0}^{2}+\text{β}_{10}\left[D\text{-}Arb\right]_{0}\left[M\right]_{0}+\text{β}_{11}\left[D\right]_{0}\left[D\text{-}Arb\right]_{0}) \end{matrix}}$ (S2)

**Diphenolase activity**

Taking into account the diphenolase activity, the S16 Fig is simplified to S17 Fig due to [M]_0_ = 0. The products formation rate is:

$\text{V}_{0}^{(Cr+P)(D)(D\text{-}Arb)}=\frac{\left[\text{α}_{2}\left[D\right]_{0}+2\text{α}_{3}\left[D\text{-}Arb\right]_{0}\right]\left[\text{E}\right]_{0}}{2(\text{β}_{5}\text{+β}_{9}\left[D\right]_{0}+\text{β}_{11}\left[D\text{-}Arb\right]_{0})}$ (S3)

$\text{V}_{0}^{(Cr+P)(D)(D\text{-}Arb)}=\frac{\frac{2\text{α}_{3}\left[D\text{-}Arb\right]_{0}\left[\text{E}\right]_{0}}{\text{β}_{9}}+\frac{\text{α}_{2}\left[D\right]_{0}\left[\text{E}\right]_{0}}{\text{β}_{9}}}{\frac{2\text{β}_{5}+2\text{β}_{11}\left[D\text{-}Arb\right]_{0}}{\text{β}_{9}}+\left[D\right]_{0}}$ (S4)

This rate correspond to the action of the enzyme on the competitive substrates.

If [D-Arb] 🡪 0, the S4 equation becomes:

$\text{V}_{0}^{Cr(D)}=\frac{\frac{\text{α}_{2}\left[D\right]_{0}\left[\text{E}\right]_{0}}{\text{β}_{9}}}{\frac{2\text{β}_{5}}{\text{β}_{9}}+\left[D\right]_{0}}=\frac{\text{V}_{\max}^{Cr(D)}\left[D\right]_{0}}{\text{K}_{M}^{D}+\left[D\right]_{0}}$ (S5)

If [D]_0_ 🡪 ∞

$\text{V}_{\max}^{Cr(D)}=\frac{\text{α}_{2}}{2\text{β}_{9}}\left[\text{E}\right]_{0}=\frac{\text{k}_{3}\text{k}_{7}}{\text{k}_{3}\text{+k}_{7}}\left[\text{E}\right]_{0}$ (S6)

**Monophenolase activity**

Taking into account that [D] = R [M], S2 equation becomes:

$\text{V}_{0}^{(Cr+P)(M)(D)(D\text{-}Arb)}=\frac{\left[\left(\text{α}_{1}R+\text{α}_{2}R^{2}\right)\left[M\right]_{0}+2\text{α}_{3}\left[D\text{-}Arb\right]_{0}R\right]\left[\text{E}\right]_{0}}{2\left[\text{β}_{4}+\text{β}_{5}R+\text{β}_{7}\text{+β}_{8}R\text{+β}_{9}\left[R\right]_{0}^{2}\left[M\right]_{0}+\left(\text{β}_{10}\text{+β}_{11}R\right)\left[D\text{-}Arb\right]_{0}\right]}$ (S7)

If [M]_0_ 🡪 ∞

$\text{V}_{0}^{(Cr)(\mathrm{M})(\mathrm{D})}=\frac{\left[\text{α}_{1}R+\text{α}_{2}R^{2}\right]\left[\text{E}\right]_{0}}{2(\text{β}_{7}\text{+β}_{8}R\text{+β}_{9}\left[R\right]_{0}^{2})}_{0}\text{=V}_{\max}^{\mathrm{Cr}(\mathrm{M})}=\frac{\text{α}_{1}}{2\text{β}_{8}}\left[\text{E}\right]_{0}=\text{k}_{5}\left[\text{E}\right]_{0}$ (S8)

When D or M 🡪 0, the activity rate of the enzyme on D-Arb corresponds to equation 7 of the manuscript.

The constants are:

α_1_ = {*K*_1_(*k*_+2_)(*k*_+3_)(*k*_+8_)(*k*_+4_)(*k*_+7_)(*k*_+5_)(*k*_-9_)(*k*_+11_)*K*_12_

+ *K*_1_(*k*_+2_)(*k*_+3_)(*k*_+8_)(*k*_+4_)(*k*_+7_)(*k*_+5_)(*k*_+10_)(*k*_+11_)*K*_12_

+ *K*_1_(*k*_+2_)(*k*_+3_)(*k*_+8_)(*k*_+4_)(*k*_-6_)(*k*_+5_)(*k*_-9_)(*k*_+11_)*K*_12_

+ *K*_1_(*k*_+2_)(*k*_+3_)(*k*_+8_)(*k*_+4_)(*k*_-6_)(*k*_+5_)(*k*_+10_)(*k*_+11_)*K*_12_}

α_2_ = {*K*_1_(*k*_+2_)(*k*_+3_)(*k*_+8_)(*k*_+6_)(*k*_+7_)(*k*_+5_)(*k*_-9_)(*k*_+11_)*K*_12_

+ *K*_1_(*k*_+2_)(*k*_+3_)(*k*_+8_)(*k*_+6_)(*k*_+7_)(*k*_+5_)(*k*_+10_)(*k*_+11_)*K*_12_

+ *K*_1_(*k*_+2_)(*k*_+3_)(*k*_+8_)(*k*_+6_)(*k*_+7_)(*k*_-4_)(*k*_-9_)(*k*_+11_)*K*_12_

+ *K*_1_(*k*_+2_)(*k*_+3_)(*k*_+8_)(*k*_+6_)(*k*_+7_)(*k*_-4_)(*k*_+10_)(*k*_+11_)*K*_12_

+ *K*_1_(*k*_+2_)(*k*_+3_)(*k*_+8_)(*k*_+6_)(*k*_+7_)(*k*_+5_)(*k*_-9_)(*k*_+11_)*K*_12_

+ *K*_1_(*k*_+2_)(*k*_+3_)(*k*_+8_)(*k*_+6_)(*k*_+7_)(*k*_+5_)(*k*_+10_)(*k*_+11_)*K*_12_

+ *K*_1_(*k*_+2_)(*k*_+3_)(*k*_+8_)(*k*_+6_)(*k*_+7_)(*k*_-4_)(*k*_-9_)(*k*_+11_)*K*_12_

+ *K*_1_(*k*_+2_)(*k*_+3_)(*k*_+8_)(*k*_+6_)(*k*_+7_)(*k*_-4_)(*k*_+10_)(*k*_+11_)*K*_12_}

α_3_ = {*K*_1_(*k*_+2_)(*k*_+3_)(*k*_+8_)(*k*_+9_)(*k*_+11_)(*k*_+7_)(*k*_+5_)(*k*_+10_)*K*_12_

+ *K*_1_(*k*_+2_)(*k*_+3_)(*k*_+8_)(*k*_+9_)(*k*_+11_)(*k*_+7_)(*k*_-4_)(*k*_+10_)*K*_12_

+ *K*_1_(*k*_+2_)(*k*_+3_)(*k*_+8_)(*k*_+9_)(*k*_+11_)(*k*_-6_)(*k*_+5_)(*k*_+10_)*K*_12_

+ *K*_1_(*k*_+2_)(*k*_+3_)(*k*_+8_)(*k*_+9_)(*k*_+11_)(*k*_-6_)(*k*_-4_)(*k*_+10_)*K*_12_]}

and

β_1_ = {*K*_1_(*k*_+2_)(*k*_+3_)(*k*_-8_)(*k*_+7_)(*k*_+5_)(*k*_-9_)(*k*_+11_)*K*_12_

+ *K*_1_(*k*_+2_)(*k*_+3_)(*k*_-8_)(*k*_+7_)(*k*_+5_)(*k*_+10_)(*k*_+11_)*K*_12_

+ *K*_1_(*k*_+2_)(*k*_+3_)(*k*_-8_)(*k*_+7_)(*k*_-4_)(*k*_-9_)(*k*_+11_)*K*_12_

+ *K*_1_(*k*_+2_)(*k*_+3_)(*k*_-8_)(*k*_+7_)(*k*_-4_)(*k*_+10_)(*k*_+11_)*K*_12_

+ *K*_1_(*k*_+2_)(*k*_+3_)(*k*_-8_)(*k*_-6_)(*k*_+5_)(*k*_-9_)(*k*_+11_)*K*_12_

+ *K*_1_(*k*_+2_)(*k*_+3_)(*k*_-8_)(*k*_-6_)(*k*_+5_)(*k*_+10_)(*k*_+11_)*K*_12_

+ *K*_1_(*k*_+2_)(*k*_+3_)(*k*_-8_)(*k*_-6_)(*k*_-4_)(*k*_-9_)(*k*_+11_)*K*_12_

+ *K*_1_(*k*_+2_)(*k*_+3_)(*k*_-8_)(*k*_-6_)(*k*_-4_)(*k*_+10_)(*k*_+11_)*K*_12_}

β_2_ = {*K*_1_(*k*_+2_)(*k*_+3_)(*k*_+4_)(*k*_+7_)(*k*_+5_)(*k*_-9_)(*k*_+11_)*K*_12_

+ *K*_1_(*k*_+2_)(*k*_+3_)(*k*_+4_)(*k*_+7_)(*k*_+5_)(*k*_+10_)(*k*_+11_)*K*_12_

+ *K*_1_(*k*_+2_)(*k*_+3_)(*k*_+4_)(*k*_-6_)(*k*_+5_)(*k*_-9_)(*k*_+11_)*K*_12_

+ *K*_1_(*k*_+2_)(*k*_+3_)(*k*_+4_)(*k*_-6_)(*k*_+5_)(*k*_+10_)(*k*_+11_)*K*_12_}

β_3_ = {*K*_1_(*k*_+2_)(*k*_+3_)(*k*_+6_)(*k*_+7_)(*k*_+5_)(*k*_-9_)(*k*_+11_)*K*_12_

+ *K*_1_(*k*_+2_)(*k*_+3_)(*k*_+6_)(*k*_+7_)(*k*_+5_)(*k*_+10_)(*k*_+11_)*K*_12_

+ *K*_1_(*k*_+2_)(*k*_+3_)(*k*_+6_)(*k*_+7_)(*k*_-4_)(*k*_-9_)(*k*_+11_)*K*_12_

+ *K*_1_(*k*_+2_)(*k*_+3_)(*k*_+6_)(*k*_+7_)(*k*_-4_)(*k*_+10_)(*k*_+11_)*K*_12_}

β_4_ = {*K*_1_(*k*_-2_)(*k*_+8_)(*k*_+4_)(*k*_+7_)(*k*_+5_)(*k*_-9_)(*k*_+11_)*K*_12_

+ *K*_1_(*k*_-2_)(*k*_+8_)(*k*_+4_)(*k*_+7_)(*k*_+5_)(*k*_+10_)(*k*_+11_)*K*_12_

+ *K*_1_(*k*_-2_)(*k*_+8_)(*k*_+4_)(*k*_-6_)(*k*_+5_)(*k*_-9_)(*k*_+11_)*K*_12_

+ *K*_1_(*k*_-2_)(*k*_+8_)(*k*_+4_)(*k*_-6_)(*k*_+5_)(*k*_+10_)(*k*_+11_)*K*_12_}

β_5_ = {*K*_1_(*k*_-2_)(*k*_+8_)(*k*_+6_)(*k*_+7_)(*k*_+5_)(*k*_-9_)(*k*_+11_)*K*_12_

+ *K*_1_(*k*_-2_)(*k*_+8_)(*k*_+6_)(*k*_+7_)(*k*_+5_)(*k*_+10_)(*k*_+11_)*K*_12_

+ *K*_1_(*k*_-2_)(*k*_+8_)(*k*_+6_)(*k*_+7_)(*k*_-4_)(*k*_-9_)(*k*_+11_)*K*_12_

+ *K*_1_(*k*_-2_)(*k*_+8_)(*k*_+6_)(*k*_+7_)(*k*_-4_)(*k*_+10_)(*k*_+11_)*K*_12_

+ *K*_1_(*k*_+3_)(*k*_+8_)(*k*_+6_)(*k*_+7_)(*k*_+5_)(*k*_-9_)(*k*_+11_)*K*_12_

+ *K*_1_(*k*_+3_)(*k*_+8_)(*k*_+6_)(*k*_+7_)(*k*_+5_)(*k*_+10_)(*k*_+11_)*K*_12_

+ *K*_1_(*k*_+3_)(*k*_+8_)(*k*_+6_)(*k*_+7_)(*k*_-4_)(*k*_-9_)(*k*_+11_)*K*_12_

+ *K*_1_(*k*_+3_)(*k*_+8_)(*k*_+6_)(*k*_+7_)(*k*_-4_)(*k*_+10_)(*k*_+11_)*K*_12_

+ *K*_1_(*k*_+2_)(*k*_+3_)(*k*_+8_)(*k*_+7_)(*k*_+5_)(*k*_-9_)(*k*_+11_)*K*_12_

+ *K*_1_(*k*_+2_)(*k*_+3_)(*k*_+8_)(*k*_+7_)(*k*_+5_)(*k*_+10_)(*k*_+11_)*K*_12_

+ *K*_1_(*k*_+2_)(*k*_+3_)(*k*_+8_)(*k*_+7_)(*k*_-4_)(*k*_-9_)(*k*_+11_)*K*_12_

+ *K*_1_(*k*_+2_)(*k*_+3_)(*k*_+8_)(*k*_+7_)(*k*_-4_)(*k*_+10_)(*k*_+11_)*K*_12_

+ *K*_1_(*k*_+2_)(*k*_+3_)(*k*_+8_)(*k*_-6_)(*k*_+5_)(*k*_-9_)(*k*_+11_)*K*_12_

+ *K*_1_(*k*_+2_)(*k*_+3_)(*k*_+8_)(*k*_-6_)(*k*_+5_)(*k*_+10_)(*k*_+11_)*K*_12_

+ *K*_1_(*k*_+2_)(*k*_+3_)(*k*_+8_)(*k*_-6_)(*k*_-4_)(*k*_-9_)(*k*_+11_)*K*_12_

+ *K*_1_(*k*_+2_)(*k*_+3_)(*k*_+8_)(*k*_-6_)(*k*_-4_)(*k*_+10_)(*k*_+11_)*K*_12_}

β_6_ = {*K*_1_(*k*_+2_)(*k*_+3_)(*k*_+9_)(*k*_+7_)(*k*_+5_)(*k*_+10_)(*k*_+11_)*K*_12_

+ *K*_1_(*k*_+2_)(*k*_+3_)(*k*_+9_)(*k*_+7_)(*k*_-4_)(*k*_+10_)(*k*_+11_)*K*_12_

+ *K*_1_(*k*_+2_)(*k*_+3_)(*k*_+9_)(*k*_-6_)(*k*_+5_)(*k*_+10_)(*k*_+11_)*K*_12_

+ *K*_1_(*k*_+2_)(*k*_+3_)(*k*_+9_)(*k*_-6_)(*k*_-4_)(*k*_+10_)(*k*_+11_)*K*_12_}

β_7_ = {(*k*_-2_)(*k*_+8_)(*k*_+4_)(*k*_+7_)(*k*_+5_)(*k*_-9_)(*k*_+11_)*K*_12_

+ (*k*_-2_)(*k*_+8_)(*k*_+4_)(*k*_+7_)(*k*_+5_)(*k*_+10_)(*k*_+11_)*K*_12_

+ (*k*_-2_)(*k*_+8_)(*k*_+4_)(*k*_-6_)(*k*_+5_)(*k*_-9_)(*k*_+11_)*K*_12_

+ (*k*_-2_)(*k*_+8_)(*k*_+4_)(*k*_-6_)(*k*_+5_)(*k*_+10_)(*k*_+11_)*K*_12_}

β_8_ = {(*k*_-2_)(*k*_+8_)(*k*_+6_)(*k*_+7_)(*k*_+5_)(*k*_-9_)(*k*_+11_)*K*_12_

+ (*k*_-2_)(*k*_+8_)(*k*_+6_)(*k*_+7_)(*k*_+5_)(*k*_+10_)(*k*_+11_)*K*_12_

+ (*k*_-2_)(*k*_+8_)(*k*_+6_)(*k*_+7_)(*k*_-4_)(*k*_-9_)(*k*_+11_)*K*_12_

+ (*k*_-2_)(*k*_+8_)(*k*_+6_)(*k*_+7_)(*k*_-4_)(*k*_+10_)(*k*_+11_)*K*_12_

+ (*k*_+3_)(*k*_+8_)(*k*_+6_)(*k*_+7_)(*k*_+5_)(*k*_-9_)(*k*_+11_)*K*_12_

+ (*k*_+3_)(*k*_+8_)(*k*_+6_)(*k*_+7_)(*k*_+5_)(*k*_+10_)(*k*_+11_)*K*_12_

+ (*k*_+3_)(*k*_+8_)(*k*_+6_)(*k*_+7_)(*k*_-4_)(*k*_-9_)(*k*_+11_)*K*_12_

+ (*k*_+3_)(*k*_+8_)(*k*_+6_)(*k*_+7_)(*k*_-4_)(*k*_+10_)(*k*_+11_)*K*_12_

+ *K*_1_(*k*_+2_)(*k*_+8_)(*k*_+4_)(*k*_+7_)(*k*_+5_)(*k*_-9_)(*k*_+11_)*K*_12_

+ *K*_1_(*k*_+2_)(*k*_+8_)(*k*_+4_)(*k*_+7_)(*k*_+5_)(*k*_+10_)(*k*_+11_)*K*_12_

+ *K*_1_(*k*_+2_)(*k*_+8_)(*k*_+4_)(*k*_-6_)(*k*_+5_)(*k*_-9_)(*k*_+11_)*K*_12_

+ *K*_1_(*k*_+2_)(*k*_+8_)(*k*_+4_)(*k*_-6_)(*k*_+5_)(*k*_+10_)(*k*_+11_)*K*_12_

+ *K*_1_(*k*_+2_)(*k*_+3_)(*k*_+8_)(*k*_+4_)(*k*_+7_)(*k*_-9_)(*k*_+11_)*K*_12_

+ *K*_1_(*k*_+2_)(*k*_+3_)(*k*_+8_)(*k*_+4_)(*k*_+7_)(*k*_+10_)(*k*_+11_)*K*_12_

+ *K*_1_(*k*_+2_)(*k*_+3_)(*k*_+8_)(*k*_+4_)(*k*_-6_)(*k*_-9_)(*k*_+11_)*K*_12_

+ *K*_1_(*k*_+2_)(*k*_+3_)(*k*_+8_)(*k*_+4_)(*k*_-6_)(*k*_+10_)(*k*_+11_)*K*_12_}

β_9_ = {*K*_1_(*k*_+2_)(*k*_+8_)(*k*_+6_)(*k*_+7_)(*k*_+5_)(*k*_-9_)(*k*_+11_)*K*_12_

+ *K*_1_(*k*_+2_)(*k*_+8_)(*k*_+6_)(*k*_+7_)(*k*_+5_)(*k*_+10_)(*k*_+11_)*K*_12_

+ *K*_1_(*k*_+2_)(*k*_+8_)(*k*_+6_)(*k*_+7_)(*k*_-4_)(*k*_-9_)(*k*_+11_)*K*_12_

+ *K*_1_(*k*_+2_)(*k*_+8_)(*k*_+6_)(*k*_+7_)(*k*_-4_)(*k*_+10_)(*k*_+11_)*K*_12_

+ *K*_1_(*k*_+2_)(*k*_+3_)(*k*_+8_)(*k*_+6_)(*k*_+5_)(*k*_-9_)(*k*_+11_)*K*_12_

+ *K*_1_(*k*_+2_)(*k*_+3_)(*k*_+8_)(*k*_+6_)(*k*_+5_)(*k*_+10_)(*k*_+11_)*K*_12_

+ *K*_1_(*k*_+2_)(*k*_+3_)(*k*_+8_)(*k*_+6_)(*k*_-4_)(*k*_-9_)(*k*_+11_)*K*_12_

+ *K*_1_(*k*_+2_)(*k*_+3_)(*k*_+8_)(*k*_+6_)(*k*_-4_)(*k*_+10_)(*k*_+11_)*K*_12_}

β_10_ = {*K*_1_(*k*_-2_)(*k*_+8_)(*k*_+4_)(*k*_+7_)(*k*_+5_)(*k*_-9_)(*k*_+11_)

+ *K*_1_(*k*_-2_)(*k*_+8_)(*k*_+4_)(*k*_+7_)(*k*_+5_)(*k*_+10_)(*k*_+11_)

+ *K*_1_(*k*_-2_)(*k*_+8_)(*k*_+4_)(*k*_-6_)(*k*_+5_)(*k*_-9_)(*k*_+11_)

+ *K*_1_(*k*_-2_)(*k*_+8_)(*k*_+4_)(*k*_-6_)(*k*_+5_)(*k*_+10_)(*k*_+11_)}

β_11_ = {*K*_1_(*k*_+2_)(*k*_+3_)(*k*_+8_)(*k*_+9_)(*k*_+7_)(*k*_+5_)(*k*_+11_)*K*_12_

+ *K*_1_(*k*_+2_)(*k*_+3_)(*k*_+8_)(*k*_+9_)(*k*_+7_)(*k*_-4_)(*k*_+11_)*K*_12_

+ *K*_1_(*k*_+2_)(*k*_+3_)(*k*_+8_)(*k*_+9_)(*k*_-6_)(*k*_+5_)(*k*_+11_)*K*_12_

+ *K*_1_(*k*_+2_)(*k*_+3_)(*k*_+8_)(*k*_+9_)(*k*_-6_)(*k*_-4_)(*k*_+11_)*K*_12_

+ *K*_1_(*k*_+2_)(*k*_+3_)(*k*_+8_)(*k*_+9_)(*k*_+7_)(*k*_+5_)(*k*_+10_)*K*_12_

+ *K*_1_(*k*_+2_)(*k*_+3_)(*k*_+8_)(*k*_+9_)(*k*_+7_)(*k*_-4_)(*k*_+10_)*K*_12_

+ *K*_1_(*k*_+2_)(*k*_+3_)(*k*_+8_)(*k*_+9_)(*k*_-6_)(*k*_+5_)(*k*_+10_)*K*_12_

+ *K*_1_(*k*_+2_)(*k*_+3_)(*k*_+8_)(*k*_+9_)(*k*_-6_)(*k*_-4_)(*k*_+10_)*K*_12_

+ *K*_1_(*k*_-2_)(*k*_+8_)(*k*_+6_)(*k*_+7_)(*k*_+5_)(*k*_-9_)(*k*_+11_)

+ *K*_1_(*k*_-2_)(*k*_+8_)(*k*_+6_)(*k*_+7_)(*k*_+5_)(*k*_+10_)(*k*_+11_)

+ *K*_1_(*k*_-2_)(*k*_+8_)(*k*_+6_)(*k*_+7_)(*k*_-4_)(*k*_-9_)(*k*_+11_)

+ *K*_1_(*k*_-2_)(*k*_+8_)(*k*_+6_)(*k*_+7_)(*k*_-4_)(*k*_+10_)(*k*_+11_)

+ *K*_1_(*k*_+3_)(*k*_+8_)(*k*_+6_)(*k*_+7_)(*k*_+5_)(*k*_-9_)(*k*_+11_)

+ *K*_1_(*k*_+3_)(*k*_+8_)(*k*_+6_)(*k*_+7_)(*k*_+5_)(*k*_+10_)(*k*_+11_)

+ *K*_1_(*k*_+3_)(*k*_+8_)(*k*_+6_)(*k*_+7_)(*k*_-4_)(*k*_-9_)(*k*_+11_)

+ *K*_1_(*k*_+3_)(*k*_+8_)(*k*_+6_)(*k*_+7_)(*k*_-4_)(*k*_+10_)(*k*_+11_)}

**References**

1. Varón-Castellanos R, Garcia-Moreno M, Garcia-Sevilla F, Ruiz-Galea MM, Garcia-Canovas F. Computerized derivation of the steady-state equations of enzyme reactions. Albacete: A5; 1995.

2. Rodriguez-Lopez JN, Ros JR, Varon R, Garcia-Canovas F. Oxygen Michaelis constants for tyrosinase. Biochem J. 1993;293(3):859-66. doi: 10.1042/bj2930859. pmid:8352753

3. Fenoll LG, Rodriguez-Lopez JN, Garcia-Molina F, Garcia-Canovas F, Tudela J. Michaelis constants of mushroom tyrosinase with respect to oxygen in the presence of monophenols and diphenols. Int J Biochem Cell Biol. 2002;34(4):332-6. doi: 10.1016/s1357-2725(01)00133-9. pmid:11854032

4. Garcia-Molina F, Hiner ANP, Fenoll LG, Rodriguez-Lopez JN, Garcia-Ruiz PA, Garcia-Canovas F, et al. Mushroom tyrosinase: Catalase activity, inhibition, and suicide inactivation. J Agric Food Chem. 2005;53(9):3702-9. doi: 10.1021/jf048340h. pmid:15853423
