# Supplementary material for: Structural and kinetic considerations on the catalysis of deoxyarbutin by tyrosinase
Source: PLoS One. 2017 Nov 14;12(11):e0187845. doi: 10.1371/journal.pone.0187845 (PMC5685642; doi:10.1371/journal.pone.0187845)
Supplement: S2 File — (DOCX) [file pone.0187845.s026.docx]

**Simulation assays**

The set of differential equations corresponding to the mechanisms of the manuscript are detailed below. The numerical integration of each set of differential equations was carried out by a computer program (WES) [[1](#_ENREF_1)], using for the rate constants, the values obtained in this work and that described in the bibliography when it was possible [[2-4](#_ENREF_2)]

- **S11, S22, S23 and S24 Figs:**

D[*E*_m_] = - *k*_12_[*E*_m_][D-Arb] + *k*_-12_[*E*_m_D-Arb] - *k*_15_[*E*_m_][H_2_O_2_] + *k*_-15_[*E*_ox_]

D[*E*_m_D-Arb] = *k*_12_[*E*_m_][D-Arb] - *k*_-12_[*E*_m_D-Arb]

D[*E*_d_] = *k-*_8_[*E*_ox_] + *k*_11_[*E*_m_D-ArbOH] - *k*_8_[*E*_d_][O_2_]

D[*E*_ox_] = *k*_8_[*E*_d_][O_2_] + *k*_-9_[*E*_ox_D-Arb] - *k*_-8_[*E*_ox_] - *k*_9_[*E*_ox_][D-Arb] + *k*_15_[*E*_m_][H_2_O_2_] - *k*_-15_[*E*_ox_]

D[*E*_ox_D-Arb] = *k*_9_[*E*_ox_][D-Arb] - *k*_-9_[*E*_ox_D-Arb] - *k*_10_[*E*_ox_D-Arb]

D[*E*_m_D-ArbOH] = *k*_10_[*E*_ox_D-Arb] - *k*_11_[*E*_m_D-ArbOH]

D[P] = *k*_11_[*E*_m_D-ArbOH]

D[O_2_] = 0

D[D-Arb] = 0

D[H_2_O_2_] = - *k*_15_[*E*_m_][H_2_O_2_] + *k*_-15_[*E*_ox_]

- **Fig 4:**

It only changes the differential equation for the [O_2_] respect from S11 Fig.

D[O_2_] = -*k*_8_[*E*_d_][B] + *k*_-8_[*E*_ox_]

- **S6 Fig:**

It only changes the differential equation for the [H_2_O_2_] respect from S11 Fig.

D[H_2_O_2_] = *k*_-15_[*E*_ox_] - *k*_15_[H_2_O_2_][*E*_m_] - *k*_16_[H_2_O_2_]

- **S8 Fig:**

D[*E*_m_] = - *k*_12_[*E*_m_][D-Arb] + *k*_-12_[*E*_m_D-Arb] - *k*_15_[*E*_m_][H_2_O_2_] + *k*_-15_[*E*_ox_] + *k*_-13_[*E*_m_D-ArbOH]

D[*E*_m_D-Arb] = *k*_12_[*E*_m_][D-Arb] - *k*_-12_[*E*_m_D-Arb]

D[*E*_d_] = *k-*_8_[*E*_ox_] + *k*_11_[*E*_m_D-ArbOH] - *k*_8_[*E*_d_][O_2_]

D[*E*_ox_] = *k*_8_[*E*_d_][O_2_] + *k*_-9_[*E*_ox_D-Arb] - *k*_-8_[*E*_ox_] - *k*_9_[*E*_ox_][D-Arb] + *k*_15_[*E*_m_][H_2_O_2_] - *k*_-15_[*E*_ox_]

D[*E*_ox_D-Arb] = *k*_9_[*E*_ox_][D-Arb] - *k*_-9_[*E*_ox_D-Arb] - *k*_10_[*E*_ox_D-Arb]

D[*E*_m_D-ArbOH] = *k*_10_[*E*_ox_D-Arb] - *k*_11_[*E*_m_D-ArbOH] - *k*_-13_[*E*_m_D-ArbOH]

D[P] = *k*_11_[*E*_m_D-ArbOH] + *k*_19_[D-ArbOH][O_2_]

D[O_2_] = 0

D[D-Arb] = 0

D[H_2_O_2_] = - *k*_15_[*E*_m_][H_2_O_2_] + *k*_-15_[*E*_ox_]

D[D-ArbOH] = *k*_-13_[*E*_m_D-ArbOH] - *k*_19_[D-ArbOH][O_2_]

- **S10 Fig:**

D[*E*_m_] = - *k*_12_[*E*_m_][D-Arb] + *k*_-12_[*E*_m_D-Arb] - *k*_15_[*E*_m_][H_2_O_2_] + *k*_-15_[*E*_ox_] + *k*_-13_[*E*_m_D-ArbOH] - *k*_13_[*E*_m_][D-ArbOH] + *k*_18_[*E*_ox_D-ArbOH]

D[*E*_m_D-Arb] = *k*_12_[*E*_m_][D-Arb] - *k*_-12_[*E*_m_D-Arb]

D[*E*_d_] = *k*_-8_[*E*_ox_] + *k*_11_[*E*_m_D-ArbOH] - *k*_8_[*E*_d_][O_2_]

D[*E*_ox_] = *k*_8_[*E*_d_][O_2_] + *k*_-9_[*E*_ox_D-Arb] - *k*_-8_[*E*_ox_] - *k*_9_[*E*_ox_][D-Arb] + *k*_15_[*E*_m_][H_2_O_2_] - *k*_-15_[*E*_ox_] - *k*_17_[*E*_ox_][D-ArbOH] + *k*_-17_[*E*_ox_D-ArbOH]

D[*E*_ox_D-Arb] = *k*_9_[*E*_ox_][D-Arb] - *k*_-9_[*E*_ox_D-Arb] - *k*_10_[*E*_ox_D-Arb]

D[*E*_m_D-ArbOH] = *k*_10_[*E*_ox_D-Arb] - *k*_11_[*E*_m_D-ArbOH] - *k*_-13_[*E*_m_D-ArbOH] + *k*_13_[*E*_m_][D-ArbOH]

D[*E*_ox_D-ArbOH] = *k*_17_[*E*_ox_][D-ArbOH] - *k*_-17_[*E*_ox_D-ArbOH] - *k*_18_[*E*_ox_D-ArbOH]

D[P] = *k*_11_[*E*_m_D-ArbOH] + *k*_18_[*E*_ox_D-ArbOH]

D[O_2_] = 0

D[D-Arb] = 0

D[H_2_O_2_] = - *k*_15_[*E*_m_][H_2_O_2_] + *k*_-15_[*E*_ox_]

D[D-ArbOH] = *k*_-13_[*E*_m_D-ArbOH] - *k*_13_[*E*_m_][D-ArbOH] - *k*_17_[*E*_ox_][D-ArbOH] + *k*_-17_[*E*_ox_D-ArbOH]

- **S18A Fig:**

D[*E*_m_M] = *k*_1_[*E*_m_][M] - *k*_-1_[*E*_m_M]

D[*E*_m_] = *k*_-2_[*E*_m_D] + *k*_-1_[*E*_m_M] + *k*_7_[*E*_ox_D] - *k*_1_[*E*_m_][M] - *k*_2_[*E*_m_][D] - *k*_12_[*E*_m_][D-Arb] + *k*_-12_[*E*_m_D-Arb]

D[*E*_m_D-Arb] = *k*_12_[*E*_m_][D-Arb] - *k*_-12_[*E*_m_D-Arb]

D[*E*_m_D] = *k*_2_[*E*_m_][D] + *k*_5_[*E*_ox_M] - *k*_-2_[*E*_m_D] - *k*_3_[*E*_m_D]

D[*E*_d_] = *k*_3_[*E*_m_D] + *k*_-8_[*E*_ox_] + *k*_11_[*E*_m_D-ArbOH] - *k*_8_[*E*_d_][O_2_]

D[*E*_ox_] = *k*_8_[*E*_d_][O_2_] + *k*_-6_[*E*_ox_D] + *k*_-9_[*E*_ox_D-Arb] + *k*_-4_[*E*_ox_M] - *k*_6_[*E*_ox_][D] - *k*_-8_[*E*_ox_] - *k*_9_[*E*_ox_][D-Arb] - *k*_4_[*E*_ox_][M]

D[*E*_ox_M] = *k*_4_[*E*_ox_][M] - *k*_-4_[*E*_ox_M] - *k*_5_[*E*_ox_M]

D[*E*_ox_D] = *k*_6_[*E*_ox_][D] - *k*_-6_[*E*_ox_D] - *k*_7_[*E*_ox_D]

D[*E*_ox_D-Arb] = *k*_9_[*E*_ox_][D-Arb] - *k*_-9_[*E*_ox_D-Arb] - *k*_10_[*E*_ox_D-Arb]

D[*E*_m_D-ArbOH] = *k*_10_[*E*_ox_D-Arb] - *k*_11_[*E*_m_D-ArbOH]

D[Q] = *k*_3_[*E*_m_D] + *k*_7_[*E*_ox_D] - *k*_14_[Q]

D[P] = *k*_11_[*E*_m_D-ArbOH]

D[O_2_] = 0

D[D] = *k*_-2_[*E*_m_D] - *k*_2_[*E*_m_][D] + *k*_-6_[*E*_ox_D] - *k*_6_[*E*_ox_][D] + 0.5*k*_14_[Q]

D[Cr] = 0.5*k*_14_[Q]

D[D-Arb] = 0

D[M] = 0

- **S18B Fig:**

D[*E*_m_] = *k*_-2_[*E*_m_D] + *k*_7_[*E*_ox_D] - *k*_2_[*E*_m_][D] - *k*_12_[*E*_m_][D-Arb] + *k*_-12_[*E*_m_D-Arb]

D[*E*_m_D-Arb] = *k*_12_[*E*_m_][D-Arb] - *k*_-12_[*E*_m_D-Arb]

D[*E*_m_D] = *k*_2_[D][*E*_m_] - *k*_-2_[*E*_m_D] - *k*_3_[*E*_m_D]

D[*E*_d_] = *k*_3_[*E*_m_D] + *k*_-8_[*E*_ox_] + *k*_11_[*E*_m_D-ArbOH] - *k*_8_[*E*_d_][O_2_]

D[*E*_ox_] = *k*_8_[*E*_d_][O_2_] + *k*_-6_[*E*_ox_D] + *k*_-9_[*E*_ox_D-Arb] - *k*_6_[*E*_ox_][D] - *k*_-8_[*E*_ox_] - *k*_9_[*E*_ox_][D-Arb]

D[*E*_ox_D] = *k*_6_[*E*_ox_][D] - *k*_-6_[*E*_ox_D] - *k*_7_[*E*_ox_D]

D[*E*_ox_D-Arb] = *k*_9_[*E*_ox_][D-Arb] - *k*_-9_[*E*_ox_D-Arb] - *k*_10_[*E*_ox_D-Arb]

D[*E*_m_D-ArbOH] = *k*_10_[*E*_ox_D-Arb] - *k*_11_[*E*_m_D-ArbOH]

D[Q] = *k*_3_[*E*_m_D] + *k*_7_[*E*_ox_D] - *k*_14_[Q]

D[P] = *k*_11_[*E*_m_D-ArbOH]

D[O_2_] = 0

D[D] = *k*_-2_[*E*_m_D] - *k*_2_[D][*E*_m_] + *k*_-6_[*E*_ox_D] - *k*_6_[*E*_ox_][D] + 0.5*k*_14_[Q]

D[Cr] = 0.5*k*_14_[Q]

D[D-Arb] = 0

**References**

1. Garcia-Sevilla F, Garrido-del Solo C, Duggleby RG, Garcia-Canovas F, Peyro R, Varon R. Use of a windows program for simulation of the progress curves of reactants and intermediates involved in enzyme-catalyzed reactions. Biosystems. 2000;54(3):151-64. doi: 10.1016/s0303-2647(99)00071-4.

2. Rodriguez-Lopez JN, Fenoll LG, Garcia-Ruiz PA, Varon R, Tudela J, Thorneley RN, et al. Stopped-flow and steady-state study of the diphenolase activity of mushroom tyrosinase. Biochemistry. 2000;39(34):10497-506. doi: 10.1021/bi000539+. pmid:10956040

3. Garcia-Molina F, Munoz JL, Varon R, Rodriguez-Lopez JN, Garcia-Canovas F, Tudela J. A review on spectrophotometric methods for measuring the monophenolase and diphenolase activities of tyrosinase. J Agric Food Chem. 2007;55(24):9739-49. doi: 10.1021/jf0712301. pmid:17958393

4. Espín JC, Varón R, Fenoll LG, Gilabert MA, García-Ruíz PA, Tudela J, et al. Kinetic characterization of the substrate specificity and mechanism of mushroom tyrosinase. Eur J Biochem. 2000;267(5):1270-9. doi: 10.1046/j.1432-1327.2000.01013.x. pmid:10691963
